# Epidemiology of Substance Use among Forced Migrants: A Global Systematic Review

**DOI:** 10.1371/journal.pone.0159134

**Published:** 2016-07-13

**Authors:** Danielle Horyniak, Jason S. Melo, Risa M. Farrell, Victoria D. Ojeda, Steffanie A. Strathdee

**Affiliations:** 1 Division of Global Public Health, University of California San Diego, La Jolla, CA, 92093, United States of America; 2 Centre for Population Health, Burnet Institute, Melbourne, VIC, 3004, Australia; 3 School of Public Health and Preventive Medicine, Monash University, Melbourne, VIC, 3004, Australia; Queensland University of Technology, AUSTRALIA

## Abstract

**Introduction:**

Forced migration is occurring at unprecedented levels. Forced migrants may be at risk for substance use for reasons including coping with traumatic experiences, co-morbid mental health disorders, acculturation challenges and social and economic inequality. This paper aimed to systematically review the literature examining substance use among forced migrants, and identify priority areas for intervention and future research.

**Methods:**

Seven medical, allied health and social science databases were searched from inception to September 2015 in accordance with PRISMA guidelines to identify original peer-reviewed articles describing any findings relating to alcohol and/or illicit drug use among refugees, internally displaced people (IDPs), asylum seekers, people displaced by disasters and deportees. A descriptive synthesis of evidence from quantitative studies was conducted, focusing primarily on studies which used validated measures of substance use. Synthesis of evidence from qualitative studies focused on identifying prominent themes relating to the contexts and consequences of substance use. Critical Appraisal Skills Programme (CASP) checklists were used to assess methodological quality of included studies.

**Results:**

Forty-four quantitative (82% cross-sectional), 16 qualitative and three mixed-methods studies were included. Ten studies were rated as high methodological quality (16%), 39 as moderate quality (62%) and 14 as low quality (22%). The majority of research was conducted among refugees, IDPs and asylum seekers (n = 55, 87%), predominantly in high-income settings. The highest-quality prevalence estimates of hazardous/harmful alcohol use ranged from 17%-36% in camp settings and 4%-7% in community settings. Few studies collected validated measures of illicit drug use. Seven studies compared substance use among forced migrants to other migrant or native-born samples. Among eight studies which conducted multivariable analysis, male sex, trauma exposure and symptoms of mental illness were commonly identified correlates of substance use.

**Conclusion:**

Our understanding of substance use among forced migrants remains limited, particularly regarding persons displaced due to disasters, development and deportation. Despite a growing body of work among refugee-background populations, few studies include refugees in low and middle-income countries, where over 80% of the global refugee population resides. Findings suggest a need to integrate substance use prevention and treatment into services offered to forced migrants, particularly in camp settings. Efforts to develop and evaluate interventions to reduce substance use and related harms are needed.

## Introduction

### Drivers and trends in forced migration

The International Organisation for Migration (IOM) defines forced migration as “a migratory movement in which an element of coercion exists, including threats to life and livelihood, whether arising from natural or man-made causes” [1]. Three main causes of forced migration are commonly considered: conflict, disaster, and development [2].

Although the number of active conflicts globally decreased from 63 in 2008 to 42 in 2014 [3], the impacts on civilian populations are intensifying, with the number of people displaced due to conflict reaching unprecedented levels. At the end of 2014, 59.5 million people were displaced as a result of violence and persecution, an increase of 8.3 million from the previous year, among the highest annual increases ever recorded [4]. This figure comprises 19.5 million refugees (people who meet the definition provided by the 1951 United Nations (UN) Convention and its 1967 Protocol), 38.2 million internally displaced persons (IDPs; persons who essentially meet the definition of a refugee but who have not crossed an internationally recognised state border [1]), and 1.8 million asylum seekers whose claims await assessment [4]. The ongoing conflict in the Syrian Arab Republic, now in its fourth year, contributed significantly to global displacement in 2014, accounting for almost 3.9 million refugees and 7.6 million IDPs, and overtaking Afghanistan as the largest refugee source country [4].

There are three traditional ‘durable solutions’ for refugees: voluntary repatriation, local integration and resettlement. Ongoing political instability and general insecurity contribute to low levels of voluntary repatriation. Resettlement demand outweighs opportunity; in 2014, just 105,000 refugees were resettled in 26 countries, with the United States (US), Canada and Australia granting permanent residence to the largest numbers [4]. As a result, most refugees remain in countries of first asylum. While many reside in formal camps, an increasing proportion live informally in urban settings [4,5].

A second key driver of forced migration globally is environmental change [6]. Weather-related natural disasters such as cyclones or floods play a role in sudden large-scale displacement due to the destruction of homes and livelihoods. Environmental change also contributes to slow-onset migration, for example, due to food insecurity and famine brought on by drought, and the impacts of rising sea levels on low-lying areas and small island states [7]. Although numbers vary drastically by year, there has been a general increasing trend in disasters and disaster-induced displacement, with 315 disaster events and 22 million people displaced due to disasters in 2013 [8,9]. With the effects of global warming predicted to increase [6], the number of environmental migrants is expected to reach 200 million by 2050 [10]. Following initial evacuation at the time of a disaster, displacees may return to their original homes, or relocate temporarily or permanently. Most disaster-induced displacees who cannot return home remain within their country of origin, and can be considered IDPs.

Development is a third key driver of forced migration. Development projects, such as infrastructure projects or the extraction of natural resources, promise significant economic development, however, these commonly require the acquisition of land which is occupied, leading to internal displacement of residents. Data on development-induced displacement are limited but it is considered a serious concern in some of the most rapidly growing economies. For example, it has been estimated that 25–50 million Indians and 40 million Chinese have been internally displaced due to development projects in the past 50 years [11,12].

Finally, deportation, the state-ordered expulsion or voluntary departure under threat of expulsion of non-citizens for breaches of immigration or criminal law, is recognised as an increasingly important form of forced migration [13]. Rates of deportations are growing, particularly in Western countries. In the US, approximately 3.7 million ‘removals’ took place from 2003–2013, with the annual numbers of deportations doubling from 211,000 to 438,000 over this period [14]. Similarly, the number of people deported from the United Kingdom reached over 40,000 in 2011, a 46% increase since 2004 [15].

### Health impacts of forced migration

There is some evidence that immigrant populations experience better health than native populations [16,17]. This ‘healthy immigrant effect’ has been attributed to both self-selection (educated, wealthy and healthy people are more likely to have opportunities to migrate) and exclusion of unhealthy migrants at immigration pre-screening. This is, however, unlikely to be the case for forced migrant populations, as forced migration is involuntary, commonly occurs on short notice, and impacts all classes of the community.

Forced migration can have diverse health impacts at all stages of the migration journey, including during transit, in countries of first asylum, and for those who are permanently resettled, in the receiving country. Health status varies across forced migration contexts. For example, IDPs may be more vulnerable to poor health than refugees due to their continued proximity to conflict zones, and limited access to services [18]. The health of forced migrants is also influenced by pre-existing health problems in the population prior to displacement, health system capacity in the location of asylum, and limited access to health services [19–21].

### Forced migration and substance use

Alcohol and illicit drug use are important causes of morbidity and mortality, accounting for 6.5% of total disability-adjusted life-years and five million deaths globally in 2010 [22]. Some evidence suggests a low prevalence of substance use among migrant populations in general [23–25], which has been attributed to the protective effects of social and cultural norms. We hypothesise that this may not be the case, however, among forced migrants, who may be particularly vulnerable to substance use for a number of reasons.

First, forced migrants have commonly witnessed and/or personally experienced pre- and post-migration stress and trauma, including loss of homes and livelihoods, violence, torture and family separation. As such, it is no surprise that the prevalence of mental health disorders, particularly depression and post-traumatic stress disorder (PTSD), are high among this population [26–28]. Comorbidity between mental health and substance use disorders has been well documented in the general population [29–31]. An emerging literature has begun to document comorbidity among forced migrant populations [32–34], who may be particularly vulnerable to substance use as a coping mechanism.

Second, forced migrants experience acculturation challenges, the process of cultural and psychological change that follows contact with a culture other than one’s own [35]. It has been hypothesised that migrants who are highly engaged in the host culture (‘assimilation’) may engage in substance use in order to adhere to mainstream norms and gain acceptance in their new communities. This may be a concern particularly in the context of resettlement in Western countries, where substance use, particularly alcohol consumption, is normalised. There is a dearth of literature on forced migrants specifically but a growing body of research, predominantly conducted among Hispanic populations in the US, has found a significant association between acculturation to dominant norms and substance use [36–38]. Acculturation is an especially important factor for younger migrants, whose experiences are compounded by intergenerational conflict, peer pressure and feeling caught ‘between cultures’ [36,39–41]. Among young people, low levels of interest in maintaining their native culture alongside low levels of participation in their new culture, often due to discrimination and exclusion, has been associated with substance use [42].

Finally, forced migrants, particularly those resettled in Western countries, commonly experience social and economic inequality, marginalisation and discrimination [43–46]. These factors have all been shown to be important determinants of health [21,47,48], and may contribute to feelings of stress and powerlessness, which may in turn contribute to substance use. A recent systematic review examining the impacts of racial discrimination on health among children and young people found positive relationships in 60% of articles examining alcohol use as an outcome and 49% of articles examining drug use as an outcome [49]. In addition, forced migrants may be exposed to illicit drugs through residence in disadvantaged neighbourhoods where alcohol and drugs may be readily available [50,51].

### Aims and significance of this review

With forced migration occurring at unprecedented levels, the potential for increases in substance-use related morbidity and mortality is concerning. Importantly, there are also substantial indirect health consequences of substance use in this vulnerable population, as it plays an important role in a number of other prevalent health conditions, including mental health disorders [28,32], gender-based violence [52,53] and infectious diseases such as HIV, hepatitis B and C and tuberculosis [54–56]. The negative consequences of substance use may also be exacerbated among forced migrant populations due to poor knowledge about substance use, stigma, and reduced access to health services [39,57–59].

Despite being an emerging issue of global health significance, comprehensive review-level data examining substance use among forced migrant populations is lacking. Two reviews were conducted more than a decade ago [60,61], and two more recent reviews have been limited in scope, with one addressing alcohol only [62], and one examining substance use more broadly but only in conflict settings [63]. The current review aims to build on this previous work by consolidating the evidence on substance use among diverse forced migrant populations across camp, community and resettlement settings, and identifying priority areas for intervention and future research.

## Methods

This review involved structured searches of peer-reviewed literature and was conducted in accordance with the Preferred Reporting Items for Systematic Reviews and Meta-Analyses (PRISMA) guidelines (S1 File) [64]. No protocol for this systematic review has been published.

### Research questions

Our review approach was guided by three overarching questions:

1. What does available research tell us about the magnitude, and risk and protective factors for substance use and related harms among forced migrants?
2. What are the contextual factors which underlie substance use among forced migrants?
3. What evidence is available to inform the design and implementation of interventions to address harmful substance use among forced migrants?

The research questions and our subsequent search strategy were developed using the SPIDER tool, which has been designed for reviews incorporating qualitative and mixed-methods literature [65]. Our SPIDER parameters were: Sample–forced migrants; Phenomenon of Interest–substance use and related harms; Design–any; Evaluation–any; Research type–any.

### Search strategy and eligibility criteria

Seven medical, allied health and social science databases (Ovid Medline, CINAHL, Ovid PsycINFO, Ovid Embase, Sociological Abstracts, International Bibliography of the Social Sciences, SocINDEX) were searched from inception to May 2015. Search terms, developed in consultation with a medical librarian, covered the key domains of forced migration and substance use (Domains S and PI of SPIDER), and were modified slightly for each database (S2 File). Articles were also identified through search updates conducted in September 2015, hand-searching reference lists of included articles and previous review papers, and contacting authors of identified conference abstracts. Archives of 15 migration, substance use and general public health conferences held between 2010 and 2014 were also searched for relevant abstracts, and corresponding authors were contacted to enquire whether any related peer-reviewed publications were in-press or recently published. One further eligible paper was identified by an anonymous reviewer during the manuscript peer-review process. Literature searching was managed using Mendeley (Mendeley Ltd, 2015).

Studies were considered eligible for inclusion in the review if they described any findings related to alcohol or illicit drug use among forced migrant populations (defined using the IOM definition [1]). Relevant findings considered included: prevalence or frequency of use, prevalence of hazardous/harmful use or dependence (including self-reported), analysis of factors associated with substance use, substance use service provision, demand or utilisation, lived experiences of forced migrants who use substances, exposure to substance use, and engagement in the production or sales of alcohol or illicit drugs. Quantitative and qualitative studies involving forced migrant populations, as well as studies involving key experts or stakeholders working with forced migrant populations were included.

Articles were excluded if they were not original peer-reviewed research, were not published in English, French or Spanish, did not clearly identify the population as forced migrants or did not provide separate results for forced migrants, or did not report any relevant findings. Multiple reports from the same study were excluded unless they reported additional relevant data.

### Screening, selection and data extraction

Following an initial screen of titles and abstracts, potentially relevant articles were selected for full text review. A random 10% of full-text articles were checked by a second reviewer and showed high inter-rater agreement on inclusion/exclusion decision (Cohen’s Kappa DH/JM: 0.82, DH/RF: 0.82).

From included articles, article publication characteristics (e.g. year of publication, journal), study procedures (e.g. study design, participant recruitment methods, data collection methods), participant characteristics (e.g. type of forced migrant population, socio-demographic characteristics) and substance use findings were extracted into a purpose-designed Microsoft Access database by JM and RF, and independently reviewed by DH.

### Quality assessment

A quality assessment was conducted using the Critical Appraisal Skills Programme (CASP) checklists for cohort studies, case-control studies and qualitative studies [66]. The checklist for cohort studies was modified for application to cross-sectional and case studies (e.g. Question 2, ‘Was the cohort recruited in an acceptable way?” was modified to ‘Was the sample recruited in an acceptable way?’, and questions regarding follow-up of participants were excluded). Quality assessment focused on assessing strengths and weaknesses of each study; a total score was calculated for each study based on relevant checklist items and then a grade of low, moderate or high was assigned through discussion between two authors (DH and JM). For mixed methods studies, separate scores were calculated for the quantitative and qualitative components, with one overall grade assigned.

### Data synthesis

Due to the heterogeneity of study designs, populations and outcome measures, a meta-analytical approach was considered inappropriate. Synthesis of evidence from quantitative studies was descriptive; results presented focus primarily on studies which used validated measures. 95% Confidence Intervals for prevalence estimates were calculated using Stata 13.1 (Statacorp LP, Texas, USA). Synthesis of evidence from qualitative studies focused on identifying prominent themes relating to the contexts and consequences of substance use.

## Results

### Study characteristics

**Fig 1:**
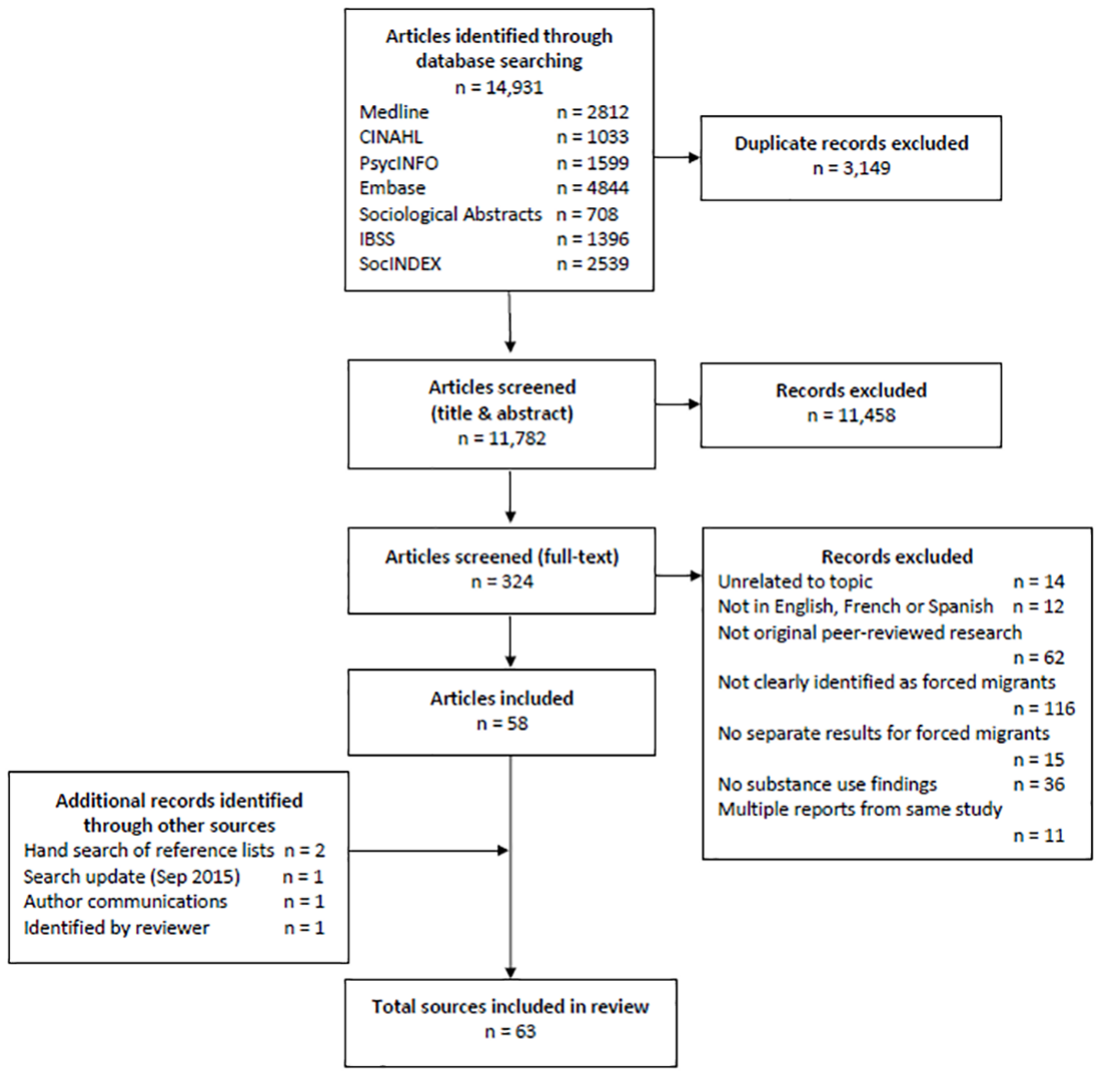
Flow chart of articles screened and selected for review.

A total of 63 relevant articles were included in the review (Fig 1). The most common reason for exclusion at full-text review was that the study population could not be clearly identified as forced migrants (n = 116, 36% of articles reviewed; Fig 1).

The majority of included articles examined substance use among refugee, IDP and asylum seeker populations (n = 55, 87%; Table 1), with a small number of studies conducted among people displaced by natural disasters (n = 4, Table 2) and deportees (n = 4, Table 3). No studies were identified among populations displaced by man-made disasters or development. Over two thirds of studies among refugees, IDPs and asylum seekers (n = 38, 69%) were conducted in high-income countries, predominantly in the US (n = 17) and Central Europe (n = 12). Of the 17 studies conducted in low and middle-income countries, 41% were conducted in Asia (n = 7), 24% in Sub-Saharan Africa (n = 4), and the remainder in Latin America, Eastern Europe and multiple country settings. All four studies involving people displaced by disasters were conducted in the US, and all four studies involving deportees were conducted in Mexico. Nine of the 51 studies which stratified by gender included samples which were exclusively male (18%), and a further 12 studies (24%) comprised at least 60% males. Only three studies focused specifically on children or young people. Just over half of all identified studies were published in the past five years.

**Table 1:** Characteristics and key findings of studies of refugees, internally displaced persons, and asylum seekers (N = 55).

Reference	Year conducted	Location	Sample	Study design, methods and measures	Observation point	Main findings	Quality assessment
***Low and middle income country settings***							
Akinyemi, 2012 [67]	--	Oru-Ijebu, Nigeria	444 adult refugee camp residents from Liberia, Sierra Leone & Togo. Mean age 34.8 years (SD 12.8), 59% male, 52% secondary educated, 24% tertiary educated. (Comparison sample: 527 adult residents of Oro community)	Cross-sectional study. Cluster sampling of camp residential blocks (refugee sample) and census areas (residential sample). Interviewer-administered questionnaire, including MINI to assess substance use.	Mean length of residence in camp: 8.6 years (SD 4.8)	Lower prevalence of alcohol abuse among the refugee sample compared with resident sample (13.5% vs. 19%), but higher prevalence of drug abuse (19.6% vs. 15.6%).	Moderate
Beckerleg, 2005 [68]	2004	Ifo, Dadaab & Hagadera, Kenya	Somali refugees who sold and/or consumed khat.	Ethnography.	--	Khat retail and use pervasive. In Ifo, 50–100 Somali refugees sell khat daily in the market. Khat chewing as a pastime and way of self-medicating feelings of hopelessness. Some evidence of alcohol and cannabis use.	Low
Ezard, 2010 [69]	2010	Thailand	1256 male Burmese refugees living in Mae-La Refugee camp. Age 15–49.	Cross-sectional feasibility study of annual screening and brief intervention for high-risk alcohol use. Opportunistic screening of outpatient clinic attendees using AUDIT(score ≥8 considered high-risk, score ≥20 considered suggestive of dependence and referred to specialist service)	--	36% positive for high-risk alcohol use and 4% had scores suggestive of alcohol dependence. Low uptake of referrals.	Moderate
Ezard, 2011 [52]	2006–2008	Kenya, Liberia, Uganda, Iran, Pakistan & Thailand	Kenya: Refugees in camp setting. 80% Sudanese. Age 17–57. 9 ethnic groups. Liberia: Returned refugees & IDPs in urban setting. Age 17–58. Uganda: IDPs in camp setting. Age 21–54. Predominantly Acholi ethnicity. Iran: Afghan refugees in urban settings. Age 16–55. Pakistan: Afghan refugees in camp and urban settings. Age 16+. Thailand: Burmese refugees in camp setting. Age 17–55.	Rapid assessments of substance use and related harms. Methods varied across settings, including observations, focus groups, and interviews with refugees, IDPs and key informants.	--	Kenya: Alcohol production and use widespread. Other substances noted: Khat, cannabis, petrol and other solvent inhalation. Alcohol used for enjoyment, socialisation and to 'kill time'. Alcohol production & sale an important source of income. Alcohol linked to GBV, mental health concerns, family disruption, diversion of household resources, risky sexual behaviour. Liberia: Alcohol and cannabis easily available, cheap and widely consumed for socialisation and relaxation. Ex-combatants considered the main sellers and users of Cannabis. Diazepam also used, particularly by combatants and other young people. Cocaine and heroin also available. Cocaine/cannabis smoking mix 'dugee' common. Uganda: Alcohol readily available and use widespread. Used for pleasure and recreation. Alcohol associated with unsafe sex, health problems, interpersonal problems, gender-based violence. Alcohol brewing a source of income for many women. For men, alcohol use linked to dispossession, alienation, idleness and loss of traditional gender roles. Cannabis also used though use hidden. Iran: Main substance opium, commonly through chasing the dragon. Heroin, 'Iranian crack' and crystal (highly concentrated forms of heroin) becoming more popular. Alcohol use rare. Cannabis (hashish) and amphetamine use reported among young people. Young male garbage pickers seen as particularly vulnerable population. Pakistan: Main substances—opium, hashish and benzodiazepines. Alcohol uncommon and mostly used by young people. Some injecting in urban but not rural areas. Limited skills, education and employment opportunities believed to promote substance use. Thailand: Alcohol cheap and readily available, mostly home-brewed. Alcohol culturally accepted response to stresses of displacement among men. Less commonly, use of yaba (amphetamine), diazepam, cough syrup, opiates, cannabis, glue inhalation.	Moderate
Ezard, 2012 [70]	2009	Thailand	636 female Burmese refugees living in Mae-La Refugee camp. Age 15–47.	Mixed methods study. Quantitative component involved all pregnant women attending the camp’s antenatal care clinic during a two week period. A single-item measure of frequency of risky high-volume drinking based on the third question from AUDIT was used to assess women’s and their reports of their male partners’ alcohol consumption. At least monthly reporting of consumption of six or more standard drinks on one occasion was considered positive. Qualitative component included interviews with 97 key informants (See Ezard, 2014 (below)).	--	Prevalence of risky alcohol use prior to pregnancy 0.2%. Reported risky alcohol use among male partners 24.4%. Strong social controls against women’s alcohol use and drinking to intoxication among males.	Moderate
Ezard, 2014 [71]	2009	Thailand	97 Burmese residents of Mae-La refugee camp with personal experience of alcohol use (either self or significant other). 68% male, 13% aged 15–20 years.	Qualitative study, using semi-structured interviews focusing on alcohol use and related harms. Recruitment through health services and chain referral.	57% displaced for less than 5 years	77% self-identified as current alcohol users. Alcohol consumed for social integration and considered to improve health and appetite. Socially accepted rules promoted drinking in moderation, particularly for women. Drinking alcohol associated with coping with life in displacement. Conversely some saw camp conditions (security, access to services, food rations) as protective against problematic alcohol use. Household economic impacts and alcohol-related violence towards women, particularly from intimate partners were key concerns.	High
Kane, 2014 [72]	2009–2013	Burundi, Rwanda, Tanzania, Chad, Djibouti, Ethiopia, Kenya, Uganda, Namibia, Zambia, Liberia, Yemen, Nepal, Bangladesh, Thailand	Attendees of primary care clinics in 90 refugee camp settings (monthly average 1.86 million refugees).	Cross-sectional study. Routinely collected outpatient clinic data used to estimated rates of visits for mental, neurological and substance use disorders. Substance use disorder defined as consumption of alcohol or another substance on a daily basis with difficulty controlling consumption.	--	Alcohol/substance use made up 1.1% of visits for all mental, neurological and substance use disorders (2.0% among males, 0.4% among females).	High
Khanani, 2010 [73]	--	Karachi & Quetta, Pakistan	556 Afghan refugees. 74% male, 43% aged 30 or younger.	Cross-sectional study. Convenience sample from antenatal clinics and free health camps.	--	23% used drugs, 7% injected drugs.	Low
Luitel, 2013 [74]	2010	Goldhap & Timai, Nepal	8021 Bhutanese aged 15 or older in two refugee camps. 49% male, mean age 35.3 years	Cross-sectional study. Census method using camp list provided by UNHCR. Interviewer-administered surveys using AUDIT to assess hazardous/harmful drinking (score ≥8) and possible alcohol dependence (score ≥20).	--	22% of men and 7% of women were current drinkers. Among current drinkers, prevalence of hazardous/harmful drinking was 23% among males and 9% among females, and the prevalence of possible dependence was 5% among males and 2% among females. In MLR, male sex, low education, history of alcohol use in the family, smoking/tobacco use, substance use and residence in Timai camp were all significantly associated with hazardous/harmful drinking.	High
Maksimovic, 2011 [75]	2000	Belgrade, Serbia	32 IDPs high school students from Kosovo. (Comparison sample: 528 high school students who had lived in Belgrade for more than 10 years)	Cross-sectional study. Census of final year students at all high schools in one central Belgrade municipality. Used self-complete anonymous questionnaire to measure past-month alcohol consumption and number of beverages consumed, past-month psychoactive substance use and number of times used. Current use defined as drank ≥1 alcoholic drink of any type in the past month, and used psychoactive substance ≥1 time in the past month.	--	56% of IDPs reported current alcohol consumption and 6% reported current psychoactive substance use. There were no significant differences in alcohol or substance use between IDP students and non-IDP students.	Moderate
Meyer, 2013 [76]	2011	Ban Mai Nai Soi, Thailand	78 Burmese (Karen) residents of Ban Mai Nai Soi refugee camp.	Qualitative study involving free listing and semi-structured interviews. Convenience sample of adult and child camp residents and purposely selected key informants.	--	Free-listing by adults and children reported alcohol consumption among both adults and children as a major problem. Alcohol associated with economic problems, violence and neglect. Alcohol use among children described as a response to stressors including poverty, adult drinking, social pressures and abuse and neglect.	Moderate
Puertas, 2006 [77]	2004	Sincelejo, Colombia	201 adult IDPs. (Comparison sample: 677 adult urban slum residents)	Cross-sectional study. Cluster random sampling of households. Self-complete questionnaire, measuring excessive alcohol consumption in the past 30 days.	--	8.5% of IDPs reported excessive alcohol consumption. There was no significant relationship between IDP status and alcohol consumption.	Moderate
Roberts, 2011 [78]	2006	Gulu and Amuru districts, Uganda	1206 IDP camp residents. 40% male, mean age 35 years.	Cross-sectional study. Multistage cluster sampling. Interviewer-administered questionnaire. Alcohol use measured using AUDIT (alcohol disorder score ≥8).	70% displaced more than 5 years	32% of men and 7% of women met the criteria for alcohol disorder. Factors significantly associated with alcohol disorder in MLR were male sex, older age, and greater cumulative trauma exposure.	High
Roberts, 2014 [79]	2011	Georgia	3600 IDPs and IDP-returnees. 35% male.	Cross-sectional study. Stratified random sampling at household level. Interviewer-administered questionnaire. Alcohol use measured using AUDIT (hazardous drinking score 8–14, harmful drinking score 15–19, dependent drinking score ≥20). Episodic heavy drinking defined by WHO as >60g pure alcohol per drinking session in the past 7 days.	--	Among current drinkers 28% of men and 1% of women reported hazardous alcohol use or more serious alcohol disorders. Among males, experiencing serious injury, and depressive symptoms were significantly associated with hazardous drinking/alcohol use disorder. Among current drinkers 12% of men and 2% of women were classified as episodic heavy drinkers. Alcohol availability was significantly associated with episodic heavy drinking.	High
Shedlin, 2014 [80]	2008–2009	Quito, Ecuador	96 Colombian refugees. 78% male.	Qualitative study combining semi-structured interviews, focus groups, ethnographic observations and media analysis. Snowball sampling used to recruit participants for individual interviews and focus groups.	--	Alcohol and drug use noted among women engaging in sex work.	Low
Streel, 2010 [81]	2009	Dadaab, Kenya and N’Zerekore, Guinea	4 camps hosting refugees primarily from Côte d’Ivoire and Liberia.	Qualitative study including observations, non-structured interviews with refugees and field workers.	--	Alcohol widely used and an important source of income. Khat commonly used among Somali refugees in Kenya. Cannabis use reported among teenagers and young adults in both sites. Substance use linked with psychological trauma, coping capacity and lack of future prospects.	Low
Zafar, 2003 [82]	2001	Quetta, Pakistan	143 Afghan refugee drug users. 100% male, median age 35 (IQR 26–41) (Comparison sample 813 Pakistani drug users:	Cross-sectional study. All new clients registering at a drug user drop-in centre. Interviewer-administered questionnaires.	--	69% of Afghan refugees currently injected drugs. 33% reported ever being in drug treatment, significantly lower than the 49% of Pakistanis who had ever been in drug treatment.	Moderate
***High income country settings***							
Arfken, 2011 [83]	--	Detroit, USA	75 Iraqi refugees. 31% male, average age 38 years. (Comparison samples: 52 non-refugee immigrants from other Arab countries; Arab-Americans in the National Survey on Drug use and Health (NSDUH); Arab/Chaldean origin participants in the Michigan Behavioural Risk Factor Surveillance System (BRFSS)).	Cross-sectional study. Participants recruited from community sites. Interviewer-administered questionnaires. Alcohol prevalence measure derived from AUDIT question “How often do you have a drink containing alcohol?”	--	13.4% reported lifetime drinking, with a higher prevalence among males than females (27.8% vs. 8.2%). Substantially lower than prevalence of lifetime drinking among Iraqi non-refugee immigrants (46.2%) and Arab-American NSDUH participants (50.8%) and past-month drinking among Arab/Chaldean BRFSS participants (45.6%). Among drinkers, refugees were newer arrivals (2.5 years) than non-refugees (5.6 years).	Moderate
Arfken, 2014 [84]	2010–2011	Southeastern Michigan, USA	298 adult Iraqi refugees. (Comparison sample: 298 non-Iraqi Arab immigrants)	Cohort study (12 months follow-up). Random sampling from refugee resettlement agencies (refugee sample) and advertisement and community presentations (non-refugee immigrant sample). Interviewer-administered questionnaires. Lifetime and past 30-day alcohol use measured at baseline, and past-year alcohol use measured at follow up.	Average time in US: 0.7 months	At baseline, the prevalence of lifetime drinking was similar among refugees and non-refugee immigrants (20.3% vs. 20.8%). Prevalence of drinking increased among refugees to 38.5% at 12-months follow-up.	Moderate
Beckwith, 2009 [85]	2000–2006	Rhode Island, USA	Cases: 52 HIV+ patients who met the UNHCR definition of refugees. 94% from Sub-Saharan Africa, mean age 34.2 years (range 21–56), 81% heterosexual. Controls: 52 HIV+ non-refugees matched on sex, age and date of initial appointment	Case-control study using routine medical records. Data collected on lifetime alcohol use and lifetime injecting drug use.	--	15% of refugee cases reported lifetime alcohol use compared with 40% of non-refugee controls. 2% of refugee cases reported lifetime injecting drug use compared with 13% of non-refugee controls.	Moderate
Bhui, 2006 [86]	--	London, UK	143 Somali refugees. 50% male, 27% aged 25 or younger, majority unemployed.	Cross-sectional study. Random sample of patients with Somali names registered with primary care services, and convenience sample recruited from community settings. Interviewer-administered questionnaires, including MINI to assess substance use.	--	Low prevalence of both alcohol and substance dependence (both 0.7%). Khat use significantly associated with mental disorder.	Moderate
Bhui, 2010 [87]	--	London, UK	180 Somali refugees. 51% male, mean age 40.4 years (range 20–88).	Cross-sectional study. Participants randomly selected from a community registry of 700 Somali people. Interviewer-administered surveys.	Mean time in UK: 8.11 years (Range: 1–16)	43% of participants used Khat on a weekly basis. Mean frequency of past-week khat use 1.3 days. Khat use not significantly associated with psychotic symptoms or anxiety or depressive symptoms.	Moderate
Brune, 2003 [34]	--	Stockholm, Sweden	N/A	Case study of 40-year old Iranian refugee seeking mental health services.	--	Use of opium, hashish and heroin to self-medicate symptoms indicative of PTSD (e.g. nightmares).	Low
Buljan, 2002 [88]	--	Vojnic, Croatia	200 adult IDPs from Bosnia & Herzegovina. 50% male, mean age 42.2 years (SD 13.6)	Cross-sectional study. Random sample from regional registry of IDPs. Structured clinical interview (DSM-IV criteria) to assess alcohol dependence.	--	Prevalence of alcohol dependence 25%. Prevalence significantly higher among those with PTSD than without (43% vs. 7%).	Low
D’Amico, 2007 [89]	2003–2005	Long Beach, USA	490 Cambodian refugees who lived under the Khmer Rouge regime. 39% male, mean age 52.2 years (SD 11.4), 72% low English proficiency.	Cross-sectional survey. Three-stage random household sampling process. Interviewer-administered questionnaire. Measures included past 30-day alcohol use, frequency of use and number of drinks consumed. Heavy alcohol drinking defined as at least one occasion in the past 30 days on which 5 or more drinks were consumed among males, or 4 or more drinks for females. Probable alcohol use disorder assessed using AUDIT (score ≥7 for women, ≥8 for men).	Mean year of immigration: 1983 (SD 3.8)	26% reported any alcohol consumption in the past 30-days. In MLR, male sex and younger age were significantly associated with any drinking in the past 30 days. 15% of male drinkers and 11% of female drinkers met AUDIT criteria for probable alcohol use disorder.	High
D’Avanzo, 1994 [90]	--	Long Beach, & Lowell, USA	120 Cambodian refugee women. Mean age 40.24 (SD 13.3). Low education and household income.	Cross-sectional study. Snowball sampling. Interviewer-administered survey, capturing alcohol and street drug use among women and their family members.	Mean time in US: 6.83 years (SD 2.72)	63% of women never used alcohol and 92% never used street drugs. Among those who consumed alcohol, common reasons for drinking were to forget troubles, and to treat emotional and physical health problems. Drinking and drug problems were reported among 7% and 8% of other family members, respectively.	Moderate
D’Avanzo, 2000 [91]	--	France and USA	155 Cambodian refugee women who had delivered a baby within the past two years. French sample older, more educated and higher language proficiency.	Cross-sectional survey. Snowball sampling. Interviewer-administered questionnaire collected data on drinking prevalence, frequency and beverages of choice.	--	34% of US participants and 15% of French women never drank. Among the French sample, 37% drank in the first trimester of pregnancy, of whom 12% drank in the third trimester. Among the US sample, 23% drank in the first trimester of pregnancy, of whom 72% drank in the third trimester.	Moderate
Dupont, 2005 [92]	1999	The Netherlands	21 asylum seekers of Somali, Afghanis, Iranian, Iraqi and Yugoslav, Palestinian, Algerian, and Guinean background. 95% male, age range 20–52.	Qualitative study using semi-structured interviews. Convenience sample recruited from asylum seeker centres.	Range: 6 months to 4.5 years.	Reasons for substance use included coping with memories and psychosocial stress, ‘killing time’ while waiting for bureaucratic processes, and boredom. Some believe Dutch drug and alcohol policy is too tolerant.	Moderate
Fu, 2010 [93]	2003–2005	New Orleans, USA	127 Vietnamese refugees aged 20–54 who arrived in the US 1975–1990. 66% male, mean age 42 (SD 4.81) (Comparison samples: 135 returnees living in Ho Chi Minh City, 447 never-leavers living in Ho Chi Minh City.	Cross-sectional study. Random sampling using registry of Vietnamese-American households (refugee sample) and multi-stage household cluster sampling (returnee and never-leaver samples). Interviewer-administered survey. Binge drinking defined as drinking five or more shots every day, and measured among male participants only.	15–30 years	16% of refugees reported binge drinking, with no significant difference between refugees, returnees and never-leavers.	Moderate
Furber, 2013 [94]	--	Wollongong, Australia	31 Burmese refugees and 10 service providers working with refugees. 61% male, age 19–65 years.	Qualitative study using focus groups and in-depth interviews.	--	Betel quid used at ceremonies and social get-togethers. Community leaders estimated that 85% of Burmese use betel quid. Betel quid chewing viewed as a more benign habit than smoking.	Low
Horyniak, 2015 [95]	2012–2013	Melbourne, Australia	16 Sudanese, Eritrean, Kenyan and Somali refugee-background men aged 18–30 years who had ever used illicit drugs.	Qualitative study using semi-structured interviews. Opportunistic sampling and agency referrals.	Median time in Australia: 10.5 years (range 6–14 years)	Alcohol consumed on a near-daily basis, with drinking to intoxication common. Key motivations for harmful drinking: to cope with pre- and post-migration trauma, to cope with boredom and marginalisation, and as a social and enjoyable experience. A range of health, social and criminal consequences of alcohol use reported. Limited engagement with services to reduce alcohol use, due to stigma, lack of support and limited knowledge of services, and perceived inability to meet needs.	High
Jenkins, 1990 [96]	--	San Francisco & Oakland, USA	215 Vietnamese refugees. 54% male, median age 35 years (range 21–78), 46% no or limited English.	Cross-sectional study. Random sampling from telephone book, and referral from refugee resettlement and support agencies. Interviewer-administered questionnaire. Measures adapted from the Behavioural Risk Factor Surveillance System. Current drinking defined as 1 or more drink in the past month, heavier drinking defined as 2 or more drinks per day in the past month, binge drinking defined as 5 or more drinks on 1 or more occasions in the past month.	Mean time in US: 7.6 years (SD 3.4)	67% of men and 18% of women current drinkers. 35% of men and 0% of women classified as binge drinkers.	Moderate
Jeon, 2008 [97]	2004	Republic of Korea	62 North Korean refugees. 55% male.	Cross-sectional study. Recruitment from a government-sponsored educational facility for North Korean refugees. Self-complete survey using the Personality Assessment Inventory, which includes scales assessing alcohol and drug problems.	Mean time since leaving North Korea: 3.3 years (SD 3.19)	Average t-score on the alcohol problems scale was 58.5 (SD 14.24) for males and 49.4 (SD 8.51) for females. Average t-score on the drug problems scale was 55.0 (SD 12.16) for males and 60.4 (SD 18.00) for females. These scores are not considered meaningfully high.	Moderate
Kluttig, 2009 [98]	--	Reichenau, Germany	N/A	Case study of an Algerian asylum seeker seeking mental health services.	--	Participant experienced cocaine and heroin use and dependence after his claim for asylum was rejected. He was treated using methadone and psychotherapy.	Low
Kozaric-Kovacic, 2000 [33]	--	Zagreb, Croatia	368 IDP camp residents from Vukovar, Slunj, and Lika regions of Croatia. 43% male.	Cross-sectional study. Random sample from camp register. Structured clinical interview. Alcohol dependence based on DSM-III-R criteria and CAGE questionnaire.	Mean time since experiencing war trauma: 30 months (SD 2.4)	61% of men and 8% of women met criteria for alcohol dependence. High prevalence of alcohol and PTSD comorbidity, particularly among men.	Moderate
Kroll, 2010 [99]	2001–2009	Minneapolis, USA	Cases: 600 Somali refugees. 47% male, 47% of males and 23% of females aged 30 or younger. Controls: 3009 non-Somali patients.	Case-control study. Patients from mental health clinic. Routine clinical data using DSM-IV-R criteria. Drug use reported on only for Somali men under age 30.	--	44% reported drug use, predominantly khat and marijuana. No association found between drug use and psychosis.	Moderate
Marshall, 2005 [100]	2003–2005	Long Beach, USA	See D’Amico, 2007 (above)	See D’Amico, 2007 (above)	See D’Amico, 2007 (above)	In MLR, year of immigration and post-migration trauma count were significantly associated with alcohol use disorder.	High
Martin, 1993 [101]	1991	Oakland,USA	8 Mien refugees from Laos who used opium. 25% male, ages 39–64.	Case series (mental health treatment setting).	6 years	Counselling and group support treatments were provided at a mental health facility. After 12 months, two patients had ceased opium use. Reasons for dropping out of treatment included family and cultural pressures	Low
McLeod, 2005 [102]	1995–1999	New Zealand	2992 newly-arrived refugees. 53% male, Most common nationalities: Iraq, Ethiopia, Somalia, Vietnam, Iran, Sudan, and Afghanistan.	Cross-sectional study. Routinely collected data from health screenings of all resettled refugees. Measured ‘drinking alcohol’, no definition provided.	At time of entry into New Zealand	4.5% drank alcohol (7.3% of males, 1.1% of females).	Low
Miremadi, 2011 [103]	2008–2009	Vancouver, Canada	68 Iraqi, Iranian, and Afghani refugees. 47% male, mean age 34.1 (SD 12.8)	Cross-sectional study. All newly-arriving refugees attending a government-sponsored intake facility invited to participate. Interviewer-administered surveys, including AUDIT (score ≥6 for women and ≥8 for men indicating hazardous alcohol use) and DUDIT.	Mean time since arrival in Canada: 7.4 days (SD 2.8)	16% of males but no females reported hazardous alcohol use. No participants recorded illicit drug use on the DUDIT.	Moderate
Mukeshimana, 2001 [104]	1999	Waterloo, USA	557 Bosnian refugees.	Cross-sectional study using routinely collected medical records.	--	20% reported drinking alcohol, of whom none reported binge drinking.	Low
Palic, 2014 [105]	2010–2011	Denmark	116 Bosnian refugees. 47% male, mean age 46.5 (SD 8.1)	Cross-sectional study. Recruitment from mental health settings. Patients with severe alcohol or drug addiction excluded. Used MCMI-III which reflects DSM-IV criteria to diagnose probable alcohol and drug dependence.	Mean time in Denmark: 16.1 years	10% of participants met criteria for probable alcohol dependence and 1% for probable drug dependence.	Moderate
Pfortmueller, 2013 [106]	2000–2012	Bern, Switzerland	3170 refugees and asylum seekers. 76% male, median age 28 (range 16–82), 49% from Africa, 24% from Middle East.	Cross-sectional study using routinely collected data from emergency department presentations.	--	7% prevalence of addiction disorder (not further specified).	Low
Portes, 1992 [107]	1986–1987	Miami, USA	952 Mariel Cuban and Haitian refugees.	Cross-sectional study. Stratified random sampling of households. Alcohol abuse/dependence measured using DSM-III criteria.	--	Prevalence of alcohol abuse/dependence 6% among Mariel Cubans and 1% among Haitians.	Moderate
Posselt, 2015 [108]	2013–2014	Adelaide, Australia	15 young refugees aged and 15 service providers working with refugee young people. 40% male, mean age 17.7 (range 12–25) Participants from Bhutan, Afghanistan and Africa.	Qualitative study using semi-structured interviews. Purposive and snowball sampling.	Mean time since migration: 4.9 years	Factors associated with mental health and substance use comorbidity included pre-migration experiences of trauma and loss, intergenerational conflict, familial separation, post-migration language and acculturation challenges, exposure to and availability of substances, maladaptive coping strategies and limited access to drug and alcohol information and services.	Moderate
Power, 2012 [109]	--	Minnesota, USA	40 Burmese (Karen) refugees	Qualitative study using focus groups and in-depth interviews	--	Drinking alcohol regularly described as an important part of social life in their community. Drinking generally reduced following migration due to cost of alcohol.	Moderate
Sabes-Figuera, 2012 [110]	2005–2006	United Kingdom, Italy, Germany	854 refugees from former Yugoslavia. 49% male. (Comparison sample: 3313 war-affected residents in 5 Balkan countries).	Cross-sectional study. In the UK, participants were recruited through community organisations and snowballing. In Italy and Germany, participants were identified from resident registers and snowballing. Interviewer-administered questionnaires, including MINI.	--	4% met criteria for substance use disorder.	Moderate
Salas-Wright, 2014 [111]	2004–2005	USA	428 refugees. 60% male. (Comparison samples: 4955 non-refugee immigrants and 29267 native-born Americans).	Cross-sectional study. Multistage cluster sampling, with oversampling of minority populations. Lifetime substance use disorders (alcohol, cannabis, cocaine, hallucinogens, amphetamines, opioids/heroin) collected using AUDASIS-IV.	--	Refugees were significantly less likely than native-born Americans to meet criteria for all substance use disorders, and significantly less likely than non-refugee immigrants to meet criteria for alcohol, cocaine, hallucinogen and opioid/heroin disorder.	High
Sandberg, 2008 [112]	2005–2006	Oslo, Norway	20 Cannabis dealers including some newly-arrived refugees.	Qualitative study using semi-structured interviews.	--	Cannabis dealing among newly-arrived refugees as a response to limited cultural capital, particularly lack of language skills and work and education opportunities.	Moderate
Steel, 2005 [113]	1999–2000	New South Wales, Australia	1161 Vietnamese refugees. 50% male. (Comparison sample: 7961 Australian-born)	Cross-sectional study. Cluster random sampling using census. Interviewer-administered surveys, with alcohol and drug use disorders collected using CIDI (DSM-IV diagnoses).	Mean time in Australia: 11.2 years (SD 5.9)	2% of males and 0% of females met criteria for alcohol use disorder. 0.8% of males and 0.2% of females met criteria for drug use disorders.	Moderate
Sundquist, 2004 [114]	1997–1999	Sweden	261,634 adult immigrants (age 25–64) born in refugee source countries. (Comparison sample: 4.2 million Swedish-born and non-refugee immigrants aged 25–64).	Cohort study. Population study using hospital admissions data. Alcohol abuse and drug abuse presentations identified using ICD classifications.	--	Age-adjusted hospital admission rates for alcohol abuse were 54.0 per 100,000 person-years for men and 35.7 per 100,000 person-years for women. Age-adjusted hospital admission rates for drug abuse were 67.8 per 100,000 person-years for men and 41.1 per 100,000 person-years for women. Among women, refugees were significantly less likely to report both alcohol and drug abuse compared with Swedish-born. Among men, refugees were significantly less likely to report alcohol abuse but significantly more likely to report drug abuse compared with Swedish-born.	Moderate
Welbel, 2013 [115]	2007–2010	180 services providing mental healthcare and support in deprived areas of: Vienna, Austria; Brussels, Belgium; Prague, Czech Republic; Paris, France; Berlin, Germany; Budapest, Hungary; Dublin, Ireland; Amsterdam, the Netherlands; Warsaw, Poland; Lisbon, Portugal; Madrid, Spain; Stockholm, Sweden; and London, England.	N/A	Cross-sectional study. Service managers completed a questionnaire-based assessment of service characteristics	N/A	Of 180 services providing substance abuse treatment, 10% provided specific programmes or services for refugee and/or asylum seeker populations.	Moderate
Westermeyer, 1989 [116]	1985–1988	Minnesota, USA	55 opioid-dependent Hmong refugees from Laos. 69% male.	Cross-sectional study. Consecutive patients recruited from a substance abuse treatment program.	--	27% used alcohol occasionally. No participants reported lifetime use of amphetamine, cannabis, cocaine, hallucinogens, inhalants, PCP or sedatives.	Low
Westermeyer, 1996 [117]	--	Minnesota, USA	57 Hmong refugees from Laos who were daily opioid users. 70% male, mean age 45.3 (SD 12.5) (Comparison sample: 80 American-born opioid (heroin) users)	Cross-sectional study. Consecutive patients recruited from a substance abuse treatment program.	--	Refugee participants commenced opioid use at a later age than American-born participants. A greater proportion of Americans had used self-help methods to reduce opiate-related problems, and significantly more had entered drug treatment.	Moderate
Yee, 1987 [118]	--	Houston, USA	840 Vietnamese refugees. 59% male, age range 18–93.	Cross-sectional study. Convenience sample from refugee service providers, multicultural organisations and telephone listings. Interviewer-administered questionnaire asking participants whether they had trouble with alcohol and taking drugs.	--	14% reported having trouble with drugs sometimes. 40% reported using alcohol to cope with sorrows or problems and 12% used drugs to cope with sorrows or problems.	Low

-- = Not reported; AUDIT = Alcohol Use Disorders Identification Test; CIDI = Composite International Diagnostic Interview DSM = Diagnostic and Statistical Manual of Mental Disorders; DUDIT: Drug Use Disorders Identification Test; ICD = International Classification of Diseases; IDP = Internally Displaced Persons; IQR = Interquartile Range: MINI = Mini-International Neuropsychiatric Interview; MLR = Multivariable Logistic Regression; N/A = Not Applicable; PTSD = Post-traumatic Stress Disorder; SD = Standard Deviation; UNHCR = United Nations High Commission for Refugees

**Table 2:** Characteristics and key findings of studies of people displaced by natural disasters (N = 4).

Reference	Year conducted	Location	Sample	Study design, methods and measures	Observation point	Main findings	Quality assessment
***High income country settings***							
Cepeda, 2010 [119]	2006–2007	Houston, USA	200 Hurricane Katrina evacuees living in Houston, who reported substance use six months prior to and/or post-Katrina, and/or being in drug treatment six months prior to Katrina. Age 18–65, 60% male, 98% African-American, 63% did not leave New Orleans before the hurricane.	Mixed methods study including interviewer-administered survey and in-depth interviews. Housing complexes drawn at random from a list of developments in two areas known to house large numbers of Katrina-evacuees.	12–24 months post-disaster	Increases in substance use reported following the disaster (29% reported increased alcohol use, 34% Marijuana, 12% Ecstasy). High incidence of ecstasy use linked to drug popularity in Houston. In MLR, leaving the city prior to the hurricane significantly associated with increased alcohol/tobacco use and increased illicit drug use. Participants who decreased drug use attributed this to displacement from drug markets and decreased access to drugs.	Moderate
Dunlap, 2009 [120]	--	Houston, USA	107 Hurricane Katrina evacuees aged ≥18 living in Houston, with a history of drug use and/or drug dealing.	Qualitative study including ethnographic observations, in-depth interviews and focus groups. Recruited through street outreach and peer-driven methods.	--	Three main factors identified which facilitated access to Houston drug market: connections with drug users/dealers; knowledge of how to locate drugs (e.g. local language); skills in navigating social scenes. Some reports of decreased drug use following displacement.	Moderate
Larrance, 2007 [121]	2006	Louisiana, USA & Mississippi, USA	366 residents from Louisiana, Mississippi and Alabama displaced after the 2005 Gulf Coast hurricane season. 65% of Louisiana respondents and 38% of Mississippi respondents were black suburban area.	Cross-sectional study. Systematic random sampling of households from Federal Emergency Management Agency trailer parks. Single household member completed an interviewer-administered questionnaire capturing information about the entire household.	Mean displacement: 246 days (SD 37.7)	14% reported they increased use and 9% reported their partner had increased use of alcohol and drugs since the hurricane. 25% reported children newly exposed to drugs and alcohol since displacement. Post-disaster substance use associated with a three-fold risk of major depressive disorder.	Moderate
Timpson, 2009 [122]	2005	Houston, USA	54 African-American Hurricane Katrina evacuees who were current crack cocaine users, living in Houston. 70% male, 85% unemployed, 25% intending to return to New Orleans. (Comparison sample: 162 African-American crack cocaine users who resided in Houston prior to the hurricane, interviewed 2002–2005)	Mixed methods study including brief quantitative survey and in-depth interviews (n = 7 displacees, n = 44 Houston residents). Street outreach and peer-driven methods.	Mean: 1.5 months	New Orleans participants were significantly more likely to use methamphetamine, marijuana and to inject drugs than Houston participants, but significantly less likely to smoke crack cocaine more than once per day. Crack cocaine reported to be available in evacuation centres and housing complexes post-displacement, and used as a coping strategy to deal with psychological effects of trauma resulting from the hurricane and subsequent displacement. Some reports of decreased drug use following displacement, with displacement seen as a ‘fresh start’.	Moderate

-- = Not reported; MLR = Multivariable logistic regression; SD = Standard deviation

**Table 3:** Characteristics and key findings of studies of deportees (N = 4).

Reference	Year conducted	Location	Sample	Study design, methods and measures	Observation point	Main findings	Quality assessment
***Low and middle income country settings***							
Brouwer, 2009 [123]	2005	Tijuana, Mexico	34 deportees who injected illicit drugs within the past month. (Comparison sample: 185 people who injected illicit drugs within the past month.)	Cross-sectional study. Respondent-driven sampling. Interviewer-administered survey.	Median time lived in Tijuana: 2 years (IQR 1–5)	Compared with other PWID, deportees were significantly more likely to injecting multiple times per day, to have ever chased heroin, and to have ever sniffed heroin, and significantly less likely to have ever smoked/inhaled methamphetamine and to have done so in the past six months. Deportees were also significantly less likely to have ever received drug treatment.	Moderate
Ojeda, 2011 [124]	2008	Tijuana, Mexico	24 male PWID with a history of deportation from the US. Mean age 36.9 years (SD 7.3). Men had lived in two US cities and experienced five deportations on average. Over half planned to return to the US.	Qualitative study. In-depth semi-structured qualitative interviews. Participants recruited from ongoing PWID cohort study, which recruited participants using respondent-driven sampling.	Mean time since most recent deportation: 7.3 years (SD 4.5)	All deportees had used illicit drugs in the US, and around half injected drugs in the US. Drug use behaviours or involvement in the drug economy contributed to deportations. Some transition to injecting by non-injectors and injection of new drugs by previous injectors post-deportation, including heroin and methamphetamine. Post-deportation drug use linked to stressors (e.g. lack of income, social networks), coping with emotional consequences of deportation (e.g. shame, loneliness), and widespread availability and low cost of drugs.	Moderate
Robertson, 2012(a) [125]	2008	Tijuana, Mexico	12 female US deportees with history of injecting drug use. Median age was 37.5 years (IQR: 32–41). Half the sample reported sex work as their primary source of income.	See Ojeda, 2011 (above)	Median: 5 years (IQR 3–10)	Deportation preceded by drug use in the US and engagement with the criminal justice system. Locating drugs was a major concern for many women immediately after deportation, with some women exchanging sex for drugs. The 4 women who rarely or never injected drugs in the USA began injecting regularly following deportation. Women described beginning to inject because of drug dependence, lack of self-control, and the influence of their social networks and neighborhoods. Although nearly all participants described wanting to "get clean" few had attended drug treatment programs in Tijuana, with financial access identified as a major barrier to treatment.	Moderate
Robertson, 2012(b) [126]	2010	Tijuana, Mexico	328 male PWID (past-month) who reported US deportation as their primary reason for moving to Tijuana. Mean age 39.3 years (SD 7.6), 74% born outside Tijuana, mean 13.7 years total US residence (SD 6.6).	Cross-sectional study. Recruited from ongoing PWID cohort study, which recruited participants using respondent-driven sampling. Interviewer-administered questionnaire.	--	16% tried new drugs post-deportation, most commonly heroin. In MLR, factors associated with new drug use post-deportation were ever being incarcerated in the US, greater number of lifetime deportations, feeling sad following most recent deportation and perceiving that one’s current lifestyle increases HIV/AIDS risk.	High

-- = Not reported; IQR = Interquartile range; PWID = person/people who inject drugs

### Study types and quality assessment

Three quarters of studies employed quantitative methods (n = 44), 16 studies utilised qualitative methods and three studies used mixed-methods. The majority of quantitative studies used cross-sectional study designs (82%). Half of the quantitative studies used probability sampling methods (n = 22, 47%) and almost two thirds (n = 28, 60%) included samples of 200 or more participants. Sixteen studies included a comparison sample of native-born or non-forced migrants. Qualitative studies employed a range of methods, including participant interviews and focus groups, key expert interviews and ethnographic methods. Ten studies were rated as high methodological quality (16%; 8 quantitative and 2 qualitative studies), 39 as moderate quality (62%) and 14 as low quality (22%).

### Synthesis of findings

#### Prevalence of alcohol use and dependence

Studies collected alcohol use prevalence estimates over the lifetime (n = 4), past year (n = 2) and past month (n = 4). Lifetime alcohol use prevalence estimates were all among refugee communities in the US and ranged from 13% among Iraqi refugees [83] to 38% among Cambodian refugee women [90]. Past-month alcohol use ranged from 26% among Cambodian refugees in the US [89] to 56% among high-school aged Serbian IDPs [75]. Studies also reported on prevalence of binge drinking, alcohol-related “problems”, having “trouble with alcohol” and “excessive alcohol consumption”, but definitions for these measures were not provided. Only one study used longitudinal methods to examine changes in alcohol use over time, finding that the prevalence of lifetime alcohol use among newly-arrived Iraqi refugees in the US increased from 20% to 39% over a 12-month period [84].

**Fig 2:**
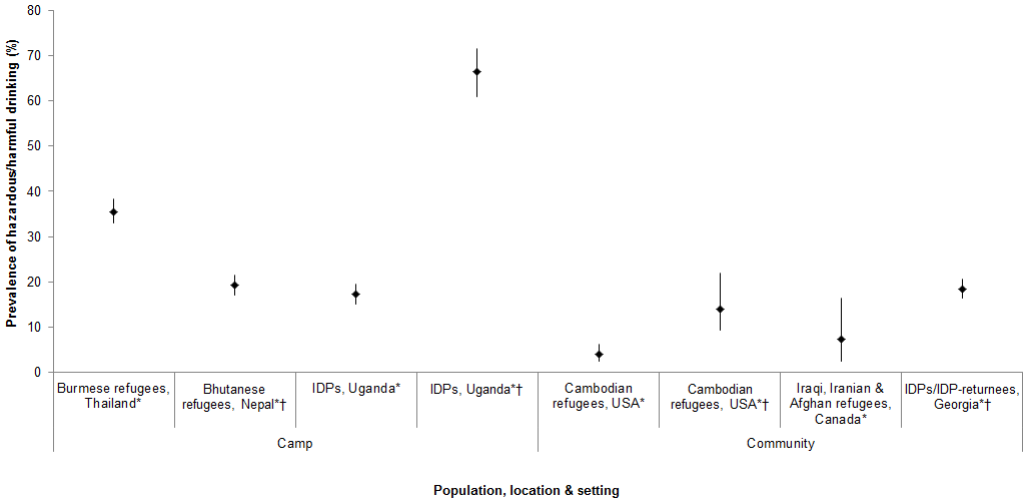
Prevalence of hazardous/harmful alcohol use among forced migrant populations, in studies using validated measures (6 studies, 8 findings).

Twelve studies measured the prevalence of hazardous/harmful alcohol use, of which six collected this information using validated measures, all using the Alcohol Use Disorders Identification Test (AUDIT [127]). In camp settings, the prevalence of hazardous/harmful alcohol use ranged from 17–36%, but was as high as 66% when measured among past-year drinkers only [78] (Fig 2). In community settings, the prevalence was 4–7% overall, and 14–19% among current drinkers. One further study used an adapted single-item measure based on the third question of the AUDIT, finding that <1% of women attending the antenatal care clinic at a refugee camp in Thailand reported ‘risky’ alcohol use [70].

**Fig 3:**
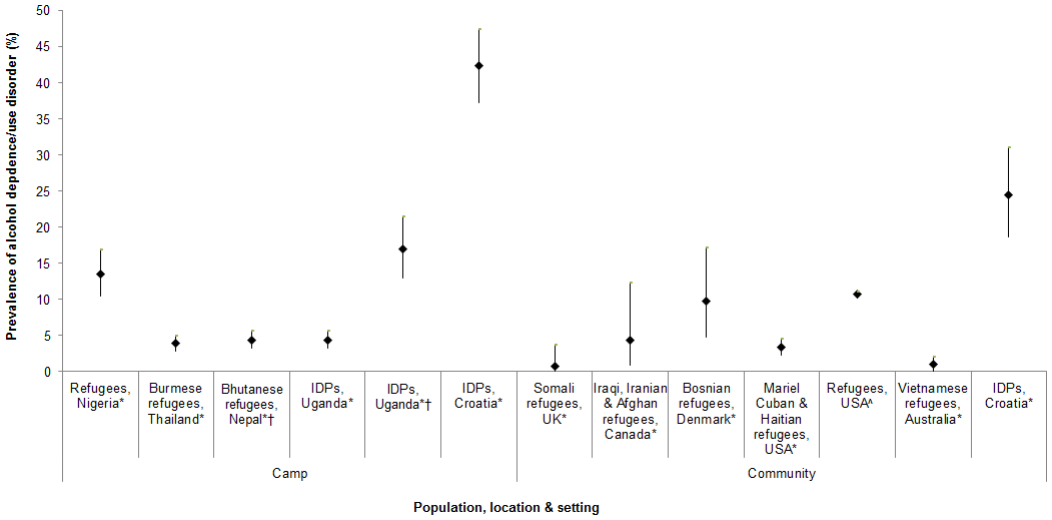
Prevalence of alcohol dependence/use disorder among forced migrant populations, among studies using validated measures (12 studies,13 findings).

Fig 3 shows the prevalence of alcohol dependence/alcohol use disorder assessed in 12 studies using validated measures (AUDIT, Diagnostic and Statistical Manual of Mental Disorders (DSM) criteria). In camp settings, the prevalence ranged from 4% among Burmese refugees in Thailand [69] to 42% among IDPs in Croatia [33]. Among IDPs in Uganda, 4% of the total sample and 17% of past-year drinkers recorded AUDIT scores of 20 or higher, suggesting a need for alcohol treatment [78]. In community settings, the prevalence of past-year alcohol dependence/use disorder ranged from less than 1% among Somali refugees in the UK [86] to 25% among IDPs in Croatia [88]. Few studies examined prevalence of alcohol use, hazardous/harmful use or dependence/use disorder stratified by age or sex.

#### Prevalence of drug use and dependence

**Fig 4:**
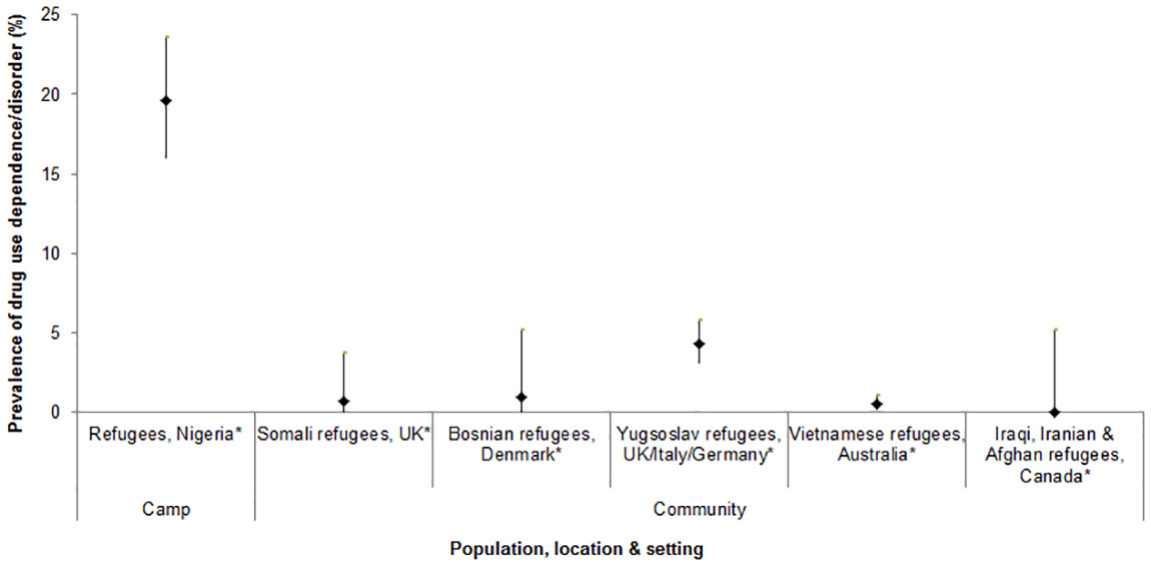
Prevalence of drug dependence/drug use disorder among forced migrant populations, among studies using validated measures (6 studies, 6 findings).

Only two studies reported on prevalence of drug use. Among a sample of Afghan refugees in Pakistan, 23% had ever used drugs and 7% had ever injected drugs [73] and among Somali refugees in the US, 44% reported any drug use [99]. No studies reported validated measures of prevalence of hazardous/harmful drug use, but six studies, all involving refugee populations, measured the prevalence of drug use dependence/disorder using validated measures (Drug Use Disorders Identification Test (DUDIT), DSM criteria; Fig 4). Prevalence was below 5% among the five studies conducted in community settings, but was 20% in the one study conducted in a camp setting [67]. None of these studies provided information about the types of drugs used among those with drug dependence.

A small number of studies reported findings related to specific drug types, most commonly cannabis, cocaine, heroin and methamphetamine. One study used the Alcohol Use Disorder and Associated Disabilities Interview Schedule-IV to measure lifetime prevalence of substance use disorders among refugees in the US, finding a prevalence of 4% for cannabis use disorder, and below 1% for cocaine, hallucinogens, amphetamines, and opioids/heroin use disorder [111]. Among disaster displacees in the US, some increased use of marijuana, ecstasy and methamphetamine was reported, with changes in patterns of drug use post-displacement linked to changes in drug availability [119,122]. Among a sample of people who inject drugs in Mexico, 16% reported trying new drugs post-deportation, most commonly heroin [126]. Cannabis, cocaine, and to a lesser extent amphetamines, were noted as emerging drugs of concern in refugee camp settings [52,81]. In Norway, an ethnographic study identified cannabis dealing as an important source of income for newly-arrived refugees who had limited cultural capital and work and educational opportunities [112].

#### Comparisons with non-forced-migrant populations

Of the 16 studies which included a comparison sample of non-forced migrants, only seven conducted statistical analysis examining differences in alcohol or drug use outcomes (Table 4). In the US, refugees were significantly less likely to report alcohol use, injecting drug use [85] and alcohol use disorder and most substance use disorders compared with non-refugees [111]. In Sweden, a population-based study found refugees were significantly less likely to experience an alcohol-related hospital admission compared with native-born, and while refugee women were significantly less likely, refugee men were significantly more likely than their non-refugee counterparts to experience a drug-related hospitalisation [114]. No statistically significant differences were found in binge drinking among Vietnamese refugees in the US, compared with returnees and never-leavers [93] or when comparing excessive alcohol consumption among IDP and non-IDP slum residents in Colombia [77].

**Table 4:** Findings from seven studies presenting analyses comparing alcohol or substance use outcomes between forced migrants and a non-forced migrant sample.

Reference & Setting	Forced migrant sample	Comparison sample	Outcome/s	Test	Results
Beckwith, 2009, USA [85]	HIV+ refugees	HIV+ non-refugees	Alcohol use; Injecting drug use	McNemar test	Refugees significantly less likely to report alcohol use compared with non-refugees: OR 0.18, 95% CI 0.06–0.52, p<0.001. Refugees significantly less likely to report engaging in IDU compared with non-refugees: OR 0.12, 95% CI 0.003–1.0, p = 0.03
Brouwer, 2009, Mexico [123]	Deportees who inject illicit drugs	Non-deportees who inject drugs	Ever chased heroin; Ever snorted/sniffed heroin; Ever smoked/inhaled methamphetamine; Smoked/inhaled methamphetamine in past 6 months; Injecting multiple times per day	Bivariate logistic regression	Deportees’ drug use behaviours differed from non-deportees, with increased odds of having ever chased heroin (OR 2.55, 95% CI 0.97–6.72, p = 0.05), ever snorted/sniffed heroin (OR 2.40, 95% CI 1.10–5.22, p<0.01), and increased odds of injecting multiple times per day (OR 5.52, 95% CI 1.62–18.8, p<0.01). Deportees had decreased odds of ever smoking/inhaling methamphetamine (OR 0.39, 95% CI 0.17–0.86, p<0.05), past 6-month methamphetamine smoking/inhalation (OR 0.38, 95% CI 0.17–0.84, p<0.05) and ever receiving drug treatment (OR 0.41, 95% CI 0.19–0.89, p<0.05)
Fu, 2010, USA [93]	Refugees	Vietnamese returnees; Vietnamese who never left Vietnam	Binge drinking (five or more shots per day)	Multivariable logistic regression, adjusted for age, marital status and occupation (analysis on males only)	No statistically significant difference in binge drinking between refugees, returnees and non-leavers: OR 0.95 for never-leavers, OR 1.15 for returnees, p>0.05 (refugees as reference group, no 95% CIs reported)
Puertas, 2006, Colombia [77]	Adult IDPs	Non-IDP urban slum residents	Excessive consumption of alcohol in past 30 days	Multivariable logistic regression, adjusted for age, sex and duration of residence in neighbourhood	No statistically significant difference in excessive alcohol consumption between IDPs and non-IDPS: OR 0.60, 95% CI 0.34–1.08, p = 0.09
Salas-Wright, 2014, USA [111]	Refugees	Native-born Americans; Non-refugee immigrants	Alcohol use disorder; Cannabis use disorder; Cocaine use disorder; Hallucinogen use disorder; Amphetamine use disorder; Heroin/opioid use disorder	Multivariable logistic regression, adjusted for age, gender, race/ethnicity, household income, education level, marital status, region of the US, urbanicity, parental antosociality and substance use history, lifetime major depressive disorder and lifetime posttraumatic stress disorder	Compared with native-born Americans, refugees were significantly less likely to report all substance use disorders: Alcohol: AOR 0.16, 95% CI 0.15–0.17, p<0.001; Cannabis: AOR 0.29, 95% CI 0.26–0.32, p<0.001; Cocaine: AOR 0.15, 95% CI 0.14–0.17, p<0.001; Hallucinogen: AOR 0.25, 95% CI 0.23–0.28, p<0.001; Amphetamine: AOR 0.20, 95% CI 0.18–0.22, p<0.001; Heroin/opioids: AOR 0.21, 95% CI 0.19–0.24, p<0.001; Compared with non-refugee immigrants, refugees were significantly less likely to report all substance use disorders, with the exceptions of cannabis and amphetamines, for which there were no statistically significant differences: Alcohol: AOR 0.44, 95% CI 0.41–0.47, p<0.001; Cannabis: AOR 1.10, 95% CI 0.93.-1.31; Cocaine: AOR 0.54, 95% CI 0.50–0.59, p<0.001; Hallucinogen: AOR 0.66, 95% CI 0.58–0.74, p<0.001; Amphetamine: AOR 0.87, 95% CI 0.74–1.03; Heroin/opioids: AOR 0.62, 95% CI 0.58–0.66, p<0.001.
Sundquist, 2004, Sweden [114]	Adult refugees	Native-born Swedes	Hospital admission rates for alcohol abuse; Hospital admission rates for drug abuse	Multivariable Cox regression, stratified by gender and adjusted for age, marital status, education and urbanisation	For both men and women, refugees were significantly less likely to experience an alcohol-related hospital admission compared with native-born Swedes: Women: HR 0.50, 95% CI 0.41–0.60; Men: HR 0.40, 95% CI 0.35–0.46. Among women, refugees were significantly less likely to experience a drug-related hospital admission compared with native-born Swedes: HR 0.79, 95% CI 0.68–0.96. Among men, refugees were significantly more likely to experience a drug-related hospital admission compared with native-born Swedes: HR 1.31, 95% CI 0.1.16–1.47
Zafar, 2009 [82]	Afghan refugee drug users	Pakistani drug users	Opiate as first drug injected; Currently injects drugs	Multivariable logistic regression, adjusted for education, homelessness, unemployment and income	There were no statistically significant differences between refugee and non-refugee drug users in opiates as first illicit drug used (AOR 1.97, 95% CI 0.97–2.44) or current injecting drug use(AOR 0.66, 95% CI 0.18–2.44)

Excludes nine studies which included a comparison sample but did not conduct statistical analysis comparing groups. AOR = Adjusted Odds Ratio; CI = Confidence Interval; HR = Hazard Ratio; OR = Odds Ratio

Two studies compared patterns of drug use and related risk behaviour between drug-using forced migrant and non-forced migrant samples. No significant differences in the proportion of participants reporting opiates as the first drug injected or reporting current injecting drug use was found between Afghan refugees and native Pakistanis [82]. Among people who inject drugs in Tijuana, Mexico, some differences in patterns of drug use were detected between deportee and non-deportee participants [123]. A noteworthy finding of this study was that deportees were significantly less likely to report ever having received drug treatment or HIV testing compared with non-deportees who inject drugs.

#### Correlates of substance use

Seven studies conducted statistical analysis to identify correlates associated with alcohol outcomes, including current/past-month drinking, increasing alcohol & tobacco use post-disaster, and hazardous/harmful drinking (Table 5). In five of these analyses, male sex was positively associated with the outcome [74,78,89,102,119], and in three trauma exposure and/or symptoms of mental illnesswere positively associated [78,79,100].

**Table 5:** Findings from seven studies presenting analyses of correlates of alcohol use outcomes.

Reference & Setting	Forced migrant sample	Outcome	Test	Results
Cepeda, 2010, USA [119]	Disaster displacees who use crack cocaine	Increase in alcohol/tobacco use post-disaster	Multivariable logistic regression	Increased alcohol/tobacco use post-disaster was significantly associated with male sex (AOR 0.47, 95% CI 0.25–0.86, p = 0.02), age 29–58 compared with age 18–28 (AOR 0.49, 95% CI 0.27–0.89, 0.02) and being a high school graduate compared with less than high school education (AOR 2.10, 95% CI 1.14–3.87, p = 0.02)
D’Amico, 2007*, USA [89]	Cambodian refugees	Any drinking past 30 days	Multivariable logistic regression	Factors associated with reduced odds of drinking in the past 30 days were older age (AOR 0.65, 95% CI 0.52–0.81, p<0.001) and female sex (AOR 0.19, 95% CI 0.11–0.33, p<0.001)
Luitel, 2013, Nepal [74]	Bhutanese refugees	Hazardous/harmful drinking (AUDIT ≥8)	Multivariable logistic regression (among current drinkers)	Factors associated with increased odds of hazardous/harmful drinking were male sex (AOR 2.81, 95% CI 1.71–4.64, p<0.001), history of alcohol use in the family (AOR 1.55, 95% CI 1.07–2.25, p<0.05), smoking/tobacco use (AOR 2.10, 95% CI 1.35–3.27, p<0.01), substance use (AOR 10.77, 95% CI 3.90–29.75, p<0.001) and residence in Timai camp (AOR 1.55, 95% CI 1.07–2.25, p<0.05). Intermediate or higher education compared with being illiterate was associated with reduced odds of hazardous/harmful drinking (AOR 0.35, 95% CI 0.16–0.75, p = 0.01).
Marshall, 2005*, USA [100]	Cambodian refugees	Probable alcohol use disorder (AUDIT ≥7 for women, ≥8 for men)	Multivariable logistic regression, adjusted for sex and pre-migration trauma count	Age was associated with reduced odds of alcohol use disorder (AOR 0.60 per decade increase, 95% CI 0.41–0.89). Increased odds of alcohol use disorder was associated with year of immigration (AOR 6.15, 95% CI 1.14–33.30) and higher post-migration trauma count (AOR 1.99, 95% CI 1.23–3.23)
McLeod, 2005, New Zealand [102]	Newly-arrived refugees	Current alcohol use	Relative risk	Male sex was significantly associated with increased risk of drinking alcohol (RR 6.87, 95% CI 3.15–14.95, p<0.001)
Roberts, 2011, Uganda [78]	IDPs	Alcohol use disorder (AUDIT ≥8)	Multivariable logistic regression, adjusted for trauma types	Factors associated with alcohol use disorder were male sex (AOR 7.21, 95% CI 4.79–10.86, p<0.001), older age (reference: 18–29; 30–39 AOR 2.32, 95% CI 1.57–3.44, p<0.001; 40–49 AOR 2.94, 95% CI 1.74–4.98, p<0.001; ≥50 AOR 4.14, 95% CI 2.62–6.52, p<0.001), and cumulative trauma exposure (reference 0–3 events; 4–7 events AOR 1.98, 95% CI 1.01–3.88, p = 0.05; 8–11 events AOR 2.00, 95% CI 1.01–3.97, p = 0.05; 12–16 events AOR 2.11, 95% CI 1.02–4.38, p = 0.04).
Roberts, 2014, Georgia [79]	IDPs and IDP-returnees	Hazardous drinking (AUDIT ≥8; current drinking men only); Episodic heavy drinking (current drinking men only)	Multivariable logistic regression; Generalised estimating equations, adjusted for age and education	Hazardous drinking was significantly associated with age ≥65 (AOR 0.16, 95% CI 0.05–0.50, p<0.001), having experienced serious injury (AOR 2.36, 95% CI 1.38–4.05, p<0.001), cumulative trauma exposure (reference no events, 2 events AOR 2.63, 95% CI 1.17–5.92, p = 0.02; 3 events AOR 1.07, 95% CI 1.07–6.67, p = 0.04; ≥4 events AOR 2.73, 95% CI 1.22–6.09, p = 0.01), and symptoms of depression (AOR 2.65, 95% CI 1.22–5.76, p = 0.01). Episodic heavy drinking was associated with being aged 30–39 and 40–49 (AOR 2.60, 95% CI 1.21–5.57, p = 0.01; AOR 2.34, 95% CI 1.12–4.85, p = 0.02) and having experienced serious injury (AOR 1.66, 95% CI 1.00–2.75, p = 0.05). In GEE, alcogenic factor (derived from density of alcohol outlets, alcohol advertising, alcohol availability and alcohol price) was significantly associated with episodic heavy drinking (AOR 1.27, 95% CI 1.01–1.59, p = 0.04)

*Same dataset, different outcomes analysed

AOR = Adjusted Odds Ratio; AUDIT = Alcohol Use Disorders Identification Test; CI = Confidence Interval; OR = Odds Ratio; RR = Relative Risk

Studies examining correlates of drug use outcomes were limited to two studies of regular drug users, one among deportees and one among people displaced by a hurricane (Table 6). Factors identified as being significantly associated with substance use outcomes (increasing substance use post-disaster and new drug use post-deportation) included experiencing greater disaster damage, greater numbers of deportations and feeling sad or depressed [119,126].

**Table 6:** Findings from two studies presenting correlates of drug use outcomes.

Reference & Setting	Forced migrant sample	Outcome	Test	Results
Cepeda, 2010, USA [119]	Disaster displaces who use crack cocaine	Increased illicit drug use post-disaster	Multivariable logistic regression	Increased illicit drug use post-disaster was significantly associated with leaving the city before Hurricane Katrina (AOR 1.83, 95% CI 1.01–3.32, p = 0.05) and high resource loss (AOR 1.99, 95% CI 1.11–3.55, p = 0.02)
Robertson, 2012(b), Mexico [126]	Deportees who inject illicit drugs	Used new drugs following most recent deportation	RDS-adjusted multivariable logistic regression	Factors independently associated with using new drugs following most recent deportation were ever being incarcerated in the US (AOR 3.96, 95% CI 1.78–8.84), increasing total number of lifetime deportations (AOR per one unit increase 1.11, 95% CI 1.03–1.20), felt sad/depressed post-deportation (AOR 2.69, 95% CI 1.41–5.14) and perceiving that one’s current lifestyle increases risk for HIV (AOR 3.91, 95% CI 2.05–7.44)

#### Interventions to address substance use

Only one intervention study was identified—a screening and brief intervention for high-risk alcohol use in a refugee camp setting in Thailand [69]. Although feasibility of screening in this setting was established, uptake of referrals to specialist treatment was low (6%).

#### Other notable findings

Substance use was commonly examined in the context of mental health. Two case studies described use of drugs including opiates, cocaine and cannabis to self-medicate symptoms of PTSD among refugees and asylum seekers [34,98], and seven qualitative studies identified coping with trauma and loss as reasons underlying the use of alcohol and drugs [52,71,81,92,95,108,122]. Three studies examined the association between khat use (a mild stimulant native to the Horn of Africa) and mental health outcomes among Somali refugees, with mixed findings [86,87,99]. One study found that post-disaster substance use was associated with a three-fold risk of major depressive disorder among hurricane displacees in the US [121]. An assessment of 180 services providing mental healthcare and substance abuse treatment across a number of European settings found that only 10% provided specific programs for refugees and/or asylum seekers [115]. Similarly, limited access to drug and alcohol information and services was identified as a key factor associated with substance use and mental health comorbidity for refugee young people in Australia [108].

Several studies focused on substances that were traditionally used in migrants’ countries of origin. Among the Burmese refugee community in Wollongong, Australia, betel quid chewing was seen as a benign social habit [94]. Similarly, khat chewing was considered a common and socially acceptable pastime among East African communities, but was also used to cope with stress and feelings of hopelessness [68,81]. Several studies examined opium use among South-East Asian refugees, finding that fewer refugees had accessed drug use treatment compared with American-born samples [117], and that family and cultural pressures were identified as reasons for discontinuing treatment [101].

Eleven studies examined the unique context of refugee camp settings. Within these studies the important role of alcohol production and sales as a source of income for these vulnerable communities was acknowledged [52,81]. Within these settings, alcohol was also implicated in a range of harms, including gender-based violence [52,71] and neglect and violence towards children [76].

## Discussion

### Key findings and implications for public health

Our global systematic review shows that despite substantial growth in research examining substance use among forced migrants in recent years, the available body of evidence remains limited. On the basis of the available evidence however, we draw attention to several findings with important implications for public health.

A major finding of this review is that there is substantial heterogeneity in patterns of substance use across included studies. In studies using validated measures, the prevalence of hazardous/harmful alcohol use ranged from 4%-36%, alcohol dependence ranged from <1%-42% and drug dependence ranged from 1%-20%. Further, among studies including comparison samples, some found lower rates of substance use among forced compared with non-forced migrant samples, some found no statistically significant differences, and one study detected associations in different directions among male and female participants. The heterogeneity in findings likely reflects regional and global differences in patterns of substance use, which may be influenced by local context factors such as availability of substances and social norms [128,129]. This level of heterogeneity limits our capacity to draw accurate conclusions about substance use among forced migrants, but supports our belief that substance use is an issue of increasing public health significance among these populations.

A particularly notable finding is that if we consider the upper limits of these estimates, as many as one in three forced migrants may be using alcohol in harmful or hazardous ways, and, when measured among current drinkers only, this estimate may be as high as two in three. Importantly, these figures may actually underestimate the true prevalence of harmful drinking as stigma has been shown to influence non-response and underreporting of substance use among culturally diverse communities [130–133]. In particular, the prevalences of both hazardous/harmful and dependent drinking identified in our review generally tended to be higher among samples in camp settings compared with community settings, suggesting that camp settings may present a particularly vulnerable risk environment for substance use. These findings indicate a need to integrate substance use prevention and treatment into services offered to forced migrants, particularly in camp settings. Currently, the Sphere Handbook, which outlines minimum standards of provision in humanitarian response, includes only a brief mention of substance use, recommending that “people have access to health services that prevent or reduce mental health problems, including minimising harm related to alcohol and drugs” [134]. We recommend that these guidelines be expanded to provide specific actions and targets for not only minimising harm in these settings, but also addressing the underlying factors which may lead to substance use. Informed by recently published World Health Organisation (WHO) recommendations for evidence-based responses to substance use in low and middle-income countries and humanitarian settings, this should include incorporating screening, brief intervention and brief motivational conversations into routine clinical practice and increasing availability of psychosocial support and medication-assisted therapies for substance use dependence and withdrawal [135,136]. It will be important for substance use services to be integrated with mental health services, and to employ a trauma-informed care perspective which focuses on respectful and empowering practices to minimise re-traumatisation [137,138].

A second key finding of this review relates to substance use among resettled forced migrants in Western settings. Although the prevalences of alcohol and illicit drug use tended to be relatively low in studies included in this review, there was substantial variation in the recorded estimates and the time points post-migration at which these measurements were captured. Given the high rates of alcohol use occurring in refugee camp settings, it is likely that substance use among newly-arriving refugees may be occurring at higher levels than what is being detected. Although current evidence-based clinical guidelines for screening and treating newly-arrived immigrants and refugees do not specifically address substance use we suggest that, consistent with the recommendations for PTSD and intimate partner violence, clinicians should be alert for signs and symptoms of substance use, and investigate further where appropriate [139]. Given another key finding of this review was that, as in the general population [128], male sex and experiences of trauma and poor mental health are associated with substance use among forced migrants, we recommend that clinicians should be particularly alert for signs of substance use among these groups.

A final key finding is that the single longitudinal study included in this systematic review detected a statistically significant increase in the prevalence of lifetime alcohol use among newly-arrived refugees in the US over a 12-month period [84], suggesting that vulnerability to substance use may increase following resettlement. This is consistent with other research has found that migrant health decreases over time due to a range of factors including resettlement challenges, acculturation and barriers to health service use [140–142]. This finding underscores the importance of maintaining contact with nearly-arrived forced migrants to monitor changes in substance use during the early post-migration period.

### Limitations of existing research

Earlier reviews of substance use among displaced persons identified methodological issues such as small sample sizes, use of non-probability sampling methods and lack of comparison groups as major limitations of existing research [62,63]. The quality of literature identified in this updated review indicates some improvement, particularly in relation to sampling methods and use of validated measures (just under one fifth of included studies were of high methodological quality), however some concerns persist.

First, the representativeness of study samples is limited. With the overwhelming focus on refugee populations, knowledge of substance use among persons displaced due to disasters, development and deportation remains extremely limited. These groups may represent a particularly vulnerable subset of forced migrants as they commonly lack the protections and supports that are afforded to refugees and IDPs under internationally recognised treaties. Moreover, current research has focused predominantly on resettled refugees in high-income settings, and findings may not be generalisable to refugee populations in low and middle-income settings, which is a major concern given that over 80% of the global refugee population resides in these settings [4]. Importantly, studies conducted in low and middle-income countries have focused almost exclusively on camp settings; as refugees/IDPs in urban settings now outnumber those in camp settings [5], this is an important group to consider in future research. In addition, across both displacement and resettlement settings, young people and women have been under-represented among refugee, IDP and asylum seeker samples studied. Young people are a particularly important sub-group as evidence shows that alcohol and illicit drug use are leading causes of morbidity and mortality among young people [143,144]. Although women generally report lower rates of substance use compared with men they are important to include in research as they are at increased risk of developing alcohol-related health consequences and are also more vulnerable to experiencing harms from others’ drinking [70,145,146]. Second, there is a dearth of studies using longitudinal methods, which limits our understanding of how substance use changes across the migration trajectory including following resettlement. Third, few studies have included appropriate control groups, making it impossible to assess how patterns of substance use differ between forced migrants, other migrant groups and native populations. Fourth, although some studies have used multivariable analysis to identify risk factors for substance use, these analyses have tended to focus on individual-level characteristics, and there has been limited consideration of potentially important structural factors such as acculturation. Finally, the paucity of intervention studies severely limits the evidence base for responding to substance use issues among forced migrant populations.

### Limitations of this review

The heterogeneity of included studies in terms of populations, settings and outcome measures precluded pooling of data in a meta-analytic manner, and makes it difficult to draw definitive conclusions. The search strategy used focused specifically on substance use. It is possible that articles broadly examining the health or mental health of forced migrants which included substance use measures but did not include substance use terms in the article title, abstract or key words may not have been identified. The review was limited to studies published in English, French, or Spanish. Although few articles were excluded due to this eligibility criteria, it is possible that findings in excluded studies were in a different direction from those included, potentially biasing the results. This review was also limited to peer-reviewed published literature as it was not feasible to conduct grey literature searches due to limited resources. Finally, a large number of sources were reviewed by a small research team in a short period of time, and although internal checks were conducted to minimise errors in data coding and entry, some unintended errors may have occurred.

### Recommendations for future research

Many important questions regarding substance use among forced migrants remain unanswered. Further research is needed to robustly assess the burden of substance use and dependence in low and middle-income countries, as well as among other sub-groups of forced migrants who have been under-studied, such as people displaced by disasters, and deportees. Importantly, as the bulk of existing research has focused on alcohol use, there is a need for additional research examining illicit drug use, including measures of individual drug types rather than unspecified ‘drug use’, and examination of transitions between different routes of drug administration. Multi-country studies which incorporate standardised recruitment, data collection and analysis across diverse settings (such as the work done on intimate partner violence in Asia and the Pacific [147]) would be an appropriate approach to producing robust data which will be informative at the site-specific level, as well as enabling effective comparisons to be made across different contexts.

In addition to measuring prevalence and identifying risk factors for substance use, it is essential to conduct research to identify factors which are protective against substance use. Drawing on the broader epidemiological literature, particularly research conducted among migrant and minority communities, factors warranting exploration include individual-level factors such as agency, resilience, and coping skills, community-level factors such as community cohesion, social network systems, and norms about substance use, environment-level factors such as availability of alcohol and illicit drugs, and policy-level factors such as rights to obtain citizenship, and legal, economic and social integration [148–151]. Better understanding of these factors may inform broad social and economic interventions which may have downstream effects on health and wellbeing [152]. Future research is also needed to explore forced migrants’ knowledge about substance use, experiences of health and social consequences of substance use, and factors affecting perceived need for, access to, and uptake of care. Together, this information will be crucial to addressing key policy objectives of the WHO in relation to the health of migrants, namely evidence-based program and policy development, and developing migrant-friendly health systems and services [152].

Future research should be designed to ensure inclusion of under-represented groups at high risk of substance use, such young people, and should employ longitudinal methods, in order to examine changes in substance use at different time points in the migration and resettlement trajectory. In particular, prospective cohort studies are crucial in identifying new-onset substance use and changes in individuals’ patterns of substance use over time. Although there are inherent challenges in conducting cohort studies with migrant populations, such as ongoing insecurity and population mobility which may impede follow-up, strategies which have had some demonstrated success include employing dynamic and adaptive methodological approaches, collaboration with established services, ongoing community engagement and utilising the capacity of information and communication technologies [153–156]. Repeated cross-sectional studies may also be valuable in resource-constrained settings such as refugee camps; although they cannot measure trajectories of substance use within individuals, they may be a meaningful way of monitoring population-level trends in substance use within a specific setting, in the same way they have been used to monitor trends in infectious disease prevalence [157,158].

Most importantly, the major gap in knowledge regarding effective, culturally competent interventions to prevent substance use and reduce related harms must be addressed. Recent research has noted that brief community-based interventions for alcohol use disorder have been shown to be effective in high-income settings [159]; explorations of how such interventions could be adapted for low and middle income settings and for substances other than alcohol would be a useful starting point. Several systematic reviews have identified family and community support as promoting resilience and improving mental health among forced migrants [160–162]; these may be important components for consideration in future interventions addressing substance use also. A potentially useful methodological approach for future intervention studies is the stepped wedge randomised controlled trial (RCT), in which groups are randomised to receive the intervention at different time points, with all groups eventually receiving the intervention. Stepped wedge RCTs are being increasingly used to address some of the logistical and ethical concerns arising in traditional RCTs (e.g. denial of a potentially effective intervention), and have been successfully implemented in low and middle-income country settings [163,164].

## Conclusions

In summary, findings from this systematic review draw attention to our limited understanding of the epidemiology of substance use among forced migrant populations, particularly among persons displaced due to disasters, development and deportation, as well as among refugees in low and middle-income countries, where over 80% of the global refugee population resides. Findings suggest a need to integrate substance use prevention and treatment into services offered to forced migrants, particularly in camp settings. Given the recent and continuing projected increases in forced migration globally, longitudinal research to improve our understanding of substance use across the migration trajectory, and efforts to develop and evaluate interventions to reduce substance use and related harms are needed.

## Supporting Information

S1 File PRISMA checklist. (DOC)

S2 File Search Strategy. (DOCX)
