# Supplementary material for: Epidemiology of Substance Use among Forced Migrants: A Global Systematic Review
Source: PLoS One. 2016 Jul 13;11(7):e0159134. doi: 10.1371/journal.pone.0159134 (PMC4943736; doi:10.1371/journal.pone.0159134)
Supplement: S2 File — (DOCX) [file pone.0159134.s002.docx]

**S2 File. Search Strategy**

**1. Medline**

Initial Search: April 21, 2015

Search Updated: September 2, 2015

| **Search 1: Forced migration** |
| --- |
| 1. **exp “Emigration and Immigration”/** 2. **exp “Transients and Migrants”/** 3. **exp Refugees/** 4. **exp Disasters/** 5. **exp War/** 6. **exp Ethnic Conflict/** 7. **exp Civil Disorders/** 8. ("forced migration" or "forced migrant" or "return migration" or "returned migrant" or returnee or repatriate*).mp. [mp=title, abstract, original title, name of substance word, subject heading word, keyword heading word, protocol supplementary concept word, rare disease supplementary concept word, unique identifier] 9. (deport* or refugee or "internally displaced" or IDP or "asylum seeker").mp. [mp=title, abstract, original title, name of substance word, subject heading word, keyword heading word, protocol supplementary concept word, rare disease supplementary concept word, unique identifier] 10. ("displaced person*" or "displaced population*" or "conflict-induced displacement" or "development-induced displacement" or "disaster-induced displacement").mp. [mp=title, abstract, original title, name of substance word, subject heading word, keyword heading word, protocol supplementary concept word, rare disease supplementary concept word, unique identifier] 11. ("ethnic conflict" or "conflict zone" or "conflict setting" or "conflict-affected" or "armed conflict" or "combat zone" or diaspora).mp. [mp=title, abstract, original title, name of substance word, subject heading word, keyword heading word, protocol supplementary concept word, rare disease supplementary concept word, unique identifier] 12. ("humanitarian entrant" or "humanitarian emergency" or "humanitarian emergencies" or "humanitarian setting" or disaster or "complex emergency" or "complex emergencies").mp. [mp=title, abstract, original title, name of substance word, subject heading word, keyword heading word, protocol supplementary concept word, rare disease supplementary concept word, unique identifier] 13. 1 OR 2 OR 3 OR 4 OR 5 OR 6 OR 7 OR 8 OR 9 OR 10 OR 11 OR 12 |
| **Search 2: Substance use** |
| 1. **exp Drinking Behavior/** 2. **exp Alcoholic Beverages/** 3. **exp Alcoholics/** 4. **exp Substance-Related Disorders/** 5. **exp Street Drugs/** 6. **exp Drug Users/** 7. **exp Cocaine/** 8. **exp Amphetamines/** 9. **exp Catha/** 10. **exp Cannabis/** 11. **exp Opium/** 12. **exp Heroin/** 13. (alcohol or "substance use" or "substance abuse" or "drug use" or "inject* drug use" or addict*).mp. [mp=title, abstract, original title, name of substance word, subject heading word, keyword heading word, protocol supplementary concept word, rare disease supplementary concept word, unique identifier] 14. ("substance-related disorder" or cocaine or amphetamine or methamphetamine or khat or qat).mp. [mp=title, abstract, original title, name of substance word, subject heading word, keyword heading word, protocol supplementary concept word, rare disease supplementary concept word, unique identifier] 15. (cannabis or marijuana or opiate or opium or heroin or "party drugs" or "club drugs").mp. [mp=title, abstract, original title, name of substance word, subject heading word, keyword heading word, protocol supplementary concept word, rare disease supplementary concept word, unique identifier] 16. ("bath salts" or ecstasy or MDMA or ketamine or hallucinogens or inhalants).mp. [mp=title, abstract, original title, name of substance word, subject heading word, keyword heading word, protocol supplementary concept word, rare disease supplementary concept word, unique identifier] 17. 14 OR 15 OR 16 OR 17 OR 18 OR 19 OR 20 OR 21 OR 22 OR 23 OR 24 OR 25 OR 26 OR 27 OR 28 OR 29 |
| 1. 28 AND 30 |

Keywords are noted in regular type, MESH (Medical subject heading) terms in bold. MESH terms were searched using the ‘explode’ function.

**2. CINAHL**

Initial Search: April 21, 2015

Search Updated: September 2, 2015

| **Search 1: Forced migration** |
| --- |
| 1. **(MM "Emigration and Immigration")** 2. **(MM "Transients and Migrants")** 3. **(MM “Refugees”)** 4. **(MH “Disasters”)** 5. **(MH “War”)** 6. **(MH “Civil Disorders”)** 7. "forced migration" or "forced migrant" or "return migration" or "returned migrant" or returnee or repatriate* 8. deport* or refugee or "internally displaced" or IDP or "asylum seeker" or "displaced person*" 9. "displaced population*" or "conflict-induced displacement" or "development-induced displacement" or "disaster-induced displacement" or "humanitarian entrant" 10. "humanitarian emergency" or "humanitarian emergencies" or "humanitarian setting" or disaster or "complex emergency" or "complex emergencies" or "ethnic conflict" 11. "conflict zone" or "conflict setting" or "conflict-affected" or "armed conflict" or "combat zone" or diaspora or "ethnic conflict" 12. 1 OR 2 OR 3 OR 4 OR 5 OR 6 OR 7 OR 8 OR 9 OR 10 OR 11 |
| **Search 2: Substance use** |
| 1. **(MH “Drinking Behavior”)** 2. **(MH “Alcoholic Beverages”)** 3. **(MH “Substance Use Disorders”)** 4. **(MH “Street Drugs”)** 5. **(MH “Substance Abusers”)** 6. **(MH “Cocaine”)** 7. **(MH Amphetamines”)** 8. **(MM “Cannabis”)** 9. **(MH “Opium”)** 10. **(MM “Heroin”)** 11. alcohol or "substance use" or "substance abuse" or "drug use" or "inject* drug use" or addict* 12. "substance-related disorder" or cocaine or amphetamine or methamphetamine or khat or qat 13. cannabis or marijuana or opiate or opium or heroin or "party drugs" 14. "club drugs" or "bath salts" or ecstasy or MDMA or ketamine or hallucinogens or inhalants 15. 13 OR 14 OR 15 OR 16 OR 17 OR 18 OR 19 OR 20 OR 21 OR 22 OR 23 OR 24 OR 25 OR 26 |
| 1. 12 AND 27 |

Keywords are noted in regular type, CINAHL subject headings in bold. MM: Searches major headings only, MH: Searches both major and minor headings. Subject headings were searched using the ‘explode’ function

**3. PsycINFO**

Initial Search: April 21, 2015

Search Updated: September 9, 2015

| **Search 1: Forced migration** |
| --- |
| 1. **exp Human Migration/** 2. **exp Immigration/** 3. **exp War/** 4. **exp Genocide/** 5. **exp Disasters/** 6. ("forced migration" or "forced migrant" or "return migration" or "returned migrant" or returnee or repatriate*).mp. [mp=title, abstract, heading word, table of contents, key concepts, original title, tests & measures] 7. (deport* or refugee or "internally displaced" or IDP or "asylum seeker").mp. [mp=title, abstract, heading word, table of contents, key concepts, original title, tests & measures] 8. ("displaced person*" or "displaced population*" or "conflict-induced displacement" or "development-induced displacement" or "disaster-induced displacement").mp. [mp=title, abstract, heading word, table of contents, key concepts, original title, tests & measures] 9. ("humanitarian entrant" or "humanitarian emergency" or "humanitarian emergencies" or "humanitarian setting" or disaster or "complex emergency" or "complex emergencies").mp. [mp=title, abstract, heading word, table of contents, key concepts, original title, tests & measures] 10. ("ethnic conflict" or "conflict zone" or "conflict setting" or "conflict-affected" or "armed conflict" or "combat zone" or diaspora).mp. [mp=title, abstract, heading word, table of contents, key concepts, original title, tests & measures] 11. 1 OR 2 OR 3 OR 4 OR 5 OR 6 OR 7 OR 8 OR 9 OR 10 |
| **Search 2: Substance use** |
| 1. **exp Drug Addiction/** 2. **exp Drug Abuse/** 3. **exp Drug Usage/** 4. **exp Alcohol Drinking Patterns/** 5. **exp Cannabis/** 6. **exp Amphetamine/** 7. **exp Hallucinogenic Drugs/** 8. **exp Heroin/** 9. (alcohol or "substance use" or "substance abuse" or "drug use" or "inject* drug use" or addict*).mp. [mp=title, abstract, heading word, table of contents, key concepts, original title, tests & measures] 10. ("substance-related disorder" or cocaine or amphetamine or methamphetamine or khat or qat).mp. [mp=title, abstract, heading word, table of contents, key concepts, original title, tests & measures] 11. (cannabis or marijuana or opiate or opium or heroin or "party drugs" or "club drugs").mp. [mp=title, abstract, heading word, table of contents, key concepts, original title, tests & measures] 12. ("bath salts" or ecstasy or MDMA or ketamine or hallucinogens or inhalants).mp. [mp=title, abstract, heading word, table of contents, key concepts, original title, tests & measures] 13. 12 OR 13 OR 14 OR 15 OR 16 OR 17 OR 18 OR 19 OR 20 OR 21 OR 22 OR 23 |
| 1. 11 AND 24 |

Keywords are noted in regular type, Index terms **in bold.** Index terms were searched using the ‘explode’ function

**4. Embase**

Initial Search: April 21, 2015

Search Updated: September 9, 2015

| **Search 1: Forced migration** |
| --- |
| 1. **exp migration/** 2. **exp emigrant/** 3. **exp immigrant/** 4. **exp refugee/** 5. **exp refugee camp/** 6. **exp disaster/** 7. **exp disaster victim/** 8. **exp ethnic conflict/** 9. **exp genocide/** 10. **exp war/** 11. ("forced migration" or "forced migrant" or "return migration" or "returned migrant" or returnee or repatriate*).mp. [mp=title, abstract, subject headings, heading word, drug trade name, original title, device manufacturer, drug manufacturer, device trade name, keyword] 12. (deport* or refugee or "internally displaced" or IDP or "asylum seeker").mp. [mp=title, abstract, subject headings, heading word, drug trade name, original title, device manufacturer, drug manufacturer, device trade name, keyword] 13. ("displaced person*" or "displaced population*" or "conflict-induced displacement" or "development-induced displacement" or "disaster-induced displacement").mp. [mp=title, abstract, subject headings, heading word, drug trade name, original title, device manufacturer, drug manufacturer, device trade name, keyword] 14. ("humanitarian entrant" or "humanitarian emergency" or "humanitarian emergencies" or "humanitarian setting" or disaster or "complex emergency" or "complex emergencies").mp. [mp=title, abstract, subject headings, heading word, drug trade name, original title, device manufacturer, drug manufacturer, device trade name, keyword] 15. ("ethnic conflict" or "conflict zone" or "conflict setting" or "conflict-affected" or "armed conflict" or "combat zone" or diaspora).mp. [mp=title, abstract, subject headings, heading word, drug trade name, original title, device manufacturer, drug manufacturer, device trade name, keyword] 16. 1 OR 2 OR 3 OR 4 OR 5 OR 6 OR 7 OR 8 OR 9 OR 10 OR 11 OR 12 OR 13 OR 14 OR 15 |
| **Search 2: Substance use** |
| 1. **exp alcohol abuse/** 2. **exp alcoholism/** 3. **exp drug abuse/** 4. **exp substance abuse/** 5. **exp substance use/** 6. **exp drug dependence/** 7. **exp cocaine/** 8. **exp amphetamine/** 9. **exp Catha edulis extract/** 10. **exp cannabis/** 11. **exp diamorphine/** 12. **exp psychedelic agent/** 13. (alcohol or "substance use" or "substance abuse" or "drug use" or "inject* drug use" or addict*).mp. [mp=title, abstract, subject headings, heading word, drug trade name, original title, device manufacturer, drug manufacturer, device trade name, keyword] 14. ("substance-related disorder" or cocaine or amphetamine or methamphetamine or khat or qat).mp. [mp=title, abstract, subject headings, heading word, drug trade name, original title, device manufacturer, drug manufacturer, device trade name, keyword] 15. (cannabis or marijuana or opiate or opium or heroin or "party drugs" or "club drugs").mp. [mp=title, abstract, subject headings, heading word, drug trade name, original title, device manufacturer, drug manufacturer, device trade name, keyword] 16. ("bath salts" or ecstasy or MDMA or ketamine or hallucinogens or inhalants).mp. [mp=title, abstract, subject headings, heading word, drug trade name, original title, device manufacturer, drug manufacturer, device trade name, keyword] 17. 17 OR 18 OR 19 OR 20 OR 21 OR 22 OR 23 OR 24 OR 25 OR 26 OR 27 OR 28 OR 29 OR 30 OR 31 OR 32 |
| 1. 16 AND 33 |

Keywords are noted in regular type, Emtree terms **in bold.** Emtree terms were searched using the ‘explode’ function

**5. Sociological Abstracts**

Initial Search: April 22, 2015

Search Updated: September 9, 2015

| **Search 1: Forced migration** |
| --- |
| 1. **Emigration** 2. **Immigration** 3. **Internal migration** 4. **Return migration** 5. **Migrants** 6. **Refugees** 7. **Asylum** 8. **Emergencies** 9. **International conflict** 10. **Ethnic conflict** 11. **International war** 12. “forced migration” 13. “forced migrant” 14. “return migration” 15. “returned migrant” 16. returnee 17. repatriate* 18. deport* 19. refugee 20. “internally displaced” 21. IDP 22. “asylum seeker” 23. “displaced person*” 24. “displaced population*” 25. “conflict-induced displacement” 26. “development-induced displacement” 27. “disaster-induced displacement” 28. “humanitarian entrant” 29. “humanitarian emergency” 30. “humanitarian emergencies” 31. “humanitarian setting” 32. disaster 33. “complex emergency” 34. “complex emergencies” 35. “ethnic conflict” 36. “conflict zone” 37. “conflict setting” 38. “conflict-affected” 39. “armed conflict” 40. “combat zone” 41. Diaspora 42. 1 OR 2 OR 3 OR 4 OR 5 OR 6 OR 7 OR 8 OR 9 OR 10 OR 11 OR 12 OR 13 OR 14 OR 15 OR 16 OR 17 OR 18 OR 19 OR 20 OR 21 OR 22 OR 23 OR 24 OR 25 OR 26 OR 27 OR 28 OR 29 OR 30 OR 31 OR 32 OR 33 OR 34 OR 35 OR 36 OR 37 OR 38 OR 39 OR 40 OR 41 |
| **Search 2: Substance use** |
| 1. **Substance abuse** 2. **Alcoholism** 3. **Alcohol use** 4. **Alcoholic beverages** 5. **Drug injection** 6. **Drug use** 7. **Drugs** 8. **Drug addiction** 9. **Cocaine** 10. **Cannabis** 11. **Marijuana** 12. **Heroin** 13. alcohol 14. “substance use” 15. “substance abuse” 16. “drug use” 17. “inject* drug use” 18. addict* 19. “substance-related disorder” 20. cocaine 21. amphetamine 22. methamphetamine 23. khat 24. qat 25. cannabis 26. marijuana 27. opiate 28. opium 29. heroin 30. “party drugs” 31. “club drugs” 32. “bath salts” 33. ecstasy 34. MDMA 35. ketamine 36. hallucinogens 37. inhalants 38. 43 OR 44 OR 45 OR 46 OR 47 OR 48 OR 49 OR 50 OR 51 OR 52 OR 53 OR 54 OR 55 OR 56 OR 57 OR 58 OR 59 OR 60 OR 61 OR 62 OR 63 OR 64 OR 65 OR 66 OR 67 OR 68 OR 69 OR 70 OR 71 OR 72 OR 73 OR 74 OR 75 OR 76 OR 77 OR 78 OR 79 |
| 1. 42 AND 80 |

Keywords are noted in regular type, subject heading terms **in bold.** Subject heading terms were searched using the ‘explode’ function

**6. International Bibliography of the Social Sciences**

Initial Search: April 24, 2015

Search Updated: September 9, 2015

| **Search 1: Forced migration** |
| --- |
| 1. **Migrants** 2. **Forced migration** 3. **Internal migration** 4. **Return migration** 5. **Refugees** 6. **Asylum seekers** 7. **Displacement** 8. **Armed conflict** 9. **Border conflicts** 10. **Interethnic conflict** 11. **International conflicts** 12. **Political conflicts** 13. **Religious conflicts** 14. **War** 15. **Disasters** 16. “forced migration” 17. “forced migrant” 18. “return migration” 19. “returned migrant” 20. returnee 21. repatriate* 22. deport* 23. refugee 24. “internally displaced” 25. IDP 26. “asylum seeker” 27. “displaced person*” 28. “displaced population*” 29. “conflict-induced displacement” 30. “development-induced displacement” 31. “disaster-induced displacement” 32. “humanitarian entrant” 33. “humanitarian emergency” 34. “humanitarian emergencies” 35. “humanitarian setting” 36. disaster 37. “complex emergency” 38. “complex emergencies” 39. “ethnic conflict” 40. “conflict zone” 41. “conflict setting” 42. “conflict-affected” 43. “armed conflict” 44. “combat zone” 45. diaspora 46. 1 OR 2 OR 3 OR 4 OR 5 OR 6 OR 7 OR 8 OR 9 OR 10 OR 11 OR 12 OR 13 OR 14 OR 15 OR 16 OR 17 OR 18 OR 19 OR 20 OR 21 OR 22 OR 23 OR 24 OR 25 OR 26 OR 27 OR 28 OR 29 OR 30 OR 31 OR 32 OR 33 OR 34 OR 35 OR 36 OR 37 OR 38 OR 39 OR 40 OR 41 OR 42 OR 43 OR 44 OR 45 |
| **Search 2: Substance use** |
| 1. **Addiction** 2. **Alcoholic beverages** 3. **Drugs** 4. **Drug addicts** 5. **Substance use** 6. alcohol 7. “substance use” 8. “substance abuse” 9. “drug use” 10. “inject* drug use” 11. addict* 12. “substance-related disorder” 13. cocaine 14. amphetamine 15. methamphetamine 16. khat 17. qat 18. cannabis 19. marijuana 20. opiate 21. opium 22. heroin 23. “party drugs” 24. “club drugs” 25. “bath salts” 26. ecstasy 27. MDMA 28. ketamine 29. hallucinogens 30. inhalants 31. 47 OR 48 OR 49 OR 50 OR 51 OR 52 OR 53 OR 54 OR 55 OR 56 OR 57 OR 58 OR 59 OR 60 OR 61 OR 62 OR 63 OR 64 OR 65 OR 66 OR 67 OR 68 OR 69 OR 70 OR 71 OR 72 OR 73 OR 74 OR 75 OR 76 |
| 1. 46 AND 77 |

Keywords are noted in regular type, Subject heading terms **in bold.** Subject heading terms were searched using the ‘explode’ function

**7. SocINDEX**

Initial Search: April 24, 2015

Search Updated: September 9, 2015

| **Search 1: Forced migration** |
| --- |
| 1. **Forced migration** 2. **Return migration** 3. **Refugees** 4. **Deportation** 5. **War** 6. **Ethnic conflict** 7. “forced migration” 8. “forced migrant” 9. “return migration” 10. “returned migrant” 11. Returnee 12. Repatriate* 13. Deport* 14. Refugee 15. “internally displaced” 16. IDP 17. “asylum seeker” 18. “displaced person*” 19. “displaced population*” 20. “conflict-induced displacement” 21. “development-induced displacement” 22. “disaster-induced displacement” 23. “humanitarian entrant” 24. “humanitarian emergency” 25. “humanitarian emergencies” 26. “humanitarian setting” 27. Disaster 28. “complex emergency” 29. “complex emergencies” 30. “ethnic conflict” 31. “conflict zone” 32. “conflict setting” 33. “conflict-affected” 34. “armed conflict” 35. “combat zone” 36. Diaspora 37. 1 OR 2 OR 3 OR 4 OR 5 OR 6 OR 7 OR 8 OR 9 OR 10 OR 11 OR 12 OR 13 OR 14 OR 15 OR 16 OR 17 OR 18 OR 19 OR 20 OR 21 OR 22 OR 23 OR 24 OR 25 OR 26 OR 27 OR 28 OR 29 OR 30 OR 31 OR 32 OR 33 OR 34 OR 35 OR 36 |
| **Search 2: Substance use** |
| 1. **Alcoholic beverages** 2. **Substance abuse** 3. **Alcoholics** 4. **Drug addicts** 5. **Drugs of abuse** 6. **Narcotics** 7. Alcohol 8. “substance use” 9. “substance abuse” 10. “drug use” 11. “inject* drug use” 12. Addict* 13. “substance-related disorder” 14. Cocaine 15. Amphetamine 16. Methamphetamine 17. Khat 18. Qat 19. Cannabis 20. Marijuana 21. Opiate 22. Heroin 23. “party drugs” 24. “club drugs” 25. “bath salts” 26. Ecstasy 27. MDMA 28. Ketamine 29. Hallucinogens 30. Inhalants 31. 38 OR 39 OR 40 OR 41 OR 42 OR 43 OR 44 OR 45 OR 46 OR 47 OR 48 OR 49 OR 50 OR 51 OR 52 OR 53 OR 54 OR 55 OR 56 OR 57 OR 58 OR 59 OR 60 OR 61 OR 62 OR 63 OR 64 OR 65 OR 66 OR 67 |
| 1. 37 AND 68 |

Keywords are noted in regular type, Subject heading terms **in bold.** Subject heading terms were searched using the ‘explode’ function
